# Single‐cell sequencing reveals potential novel insights into appendage‐patterning and joint‐development in a spider

**DOI:** 10.1002/dvdy.70069

**Published:** 2025-08-07

**Authors:** Brenda I. Medina‐Jiménez, Graham E. Budd, Ralf Janssen

**Affiliations:** ^1^ Department of Earth Sciences, Palaeobiology Uppsala University Uppsala Sweden; ^2^ Department of Zoology, Zoologiska institutionen: Populationsgenetik Stockholm University Stockholm Sweden

**Keywords:** appendage development, appendage evolution, Arthropoda, joint development

## Abstract

**Background:**

Jointed appendages represent one of the key innovations of arthropods, and thus understanding the development and evolution of these structures is important for the understanding of the evolutionary success of Arthropoda. In this paper, we analyze a cell cluster that was identified in a previous single‐cell sequencing (SCS) experiment on embryos of the spider *Parasteatoda tepidariorum*. This cell cluster is characterized by marker genes that suggest a role in appendage patterning and joint development.

**Results:**

We analyzed the expression profiles of these marker genes showing that they are expressed in the developing appendages and in a pattern that suggests a potential function during joint development. Several of the investigated genes represent new and unexpected factors such as *dysfusion* (*dysf*), *spätzle3* (*spz3*), *seven‐up* (*svp*). In order to study their evolutionary origin, we also investigated orthologs of the identified appendage‐patterning genes in the harvestman *Phalangium opilio*, a distantly related chelicerate.

**Conclusion:**

Our work highlights the usefulness of SCS experiments for the identification of potential new genetic factors that are involved in specific developmental processes. The current data provide potential new insights into the gene regulatory networks that underlie arthropod joint development.

## Key Findings

- Single‐cell‐sequencing reveals potential new factors of arthropod joint‐development and appendage patterning Single‐cell‐sequencing overcomes the conceptual restrictions of the classical candidate gene approach Comparative gene expression provides insight into the evolutionary history of potentially novel joint‐ and appendage‐patterning genes.

## 1 INTRODUCTION

The jointed (segmented) appendages of arthropods represent one of the key innovations of arthropods. The ability to modify single segments or the number of segments in the appendages without compromising the overall architecture and functionality of a given appendage likely allowed the arthropods to quickly adapt to environmental changes during the course of their evolution that began more than 500 Mya ago in the Cambrian.^1^, ^2^, ^3^, ^4^ While the stem group ancestors of arthropods likely possessed an almost uniformly segmented body in which each body segment was equipped with a pair of locomotory appendages, during their evolution, anterior segments successively became modified and specialized for perception of the environment and food processing.^3^, ^5^, ^6^

**FIGURE 1:**
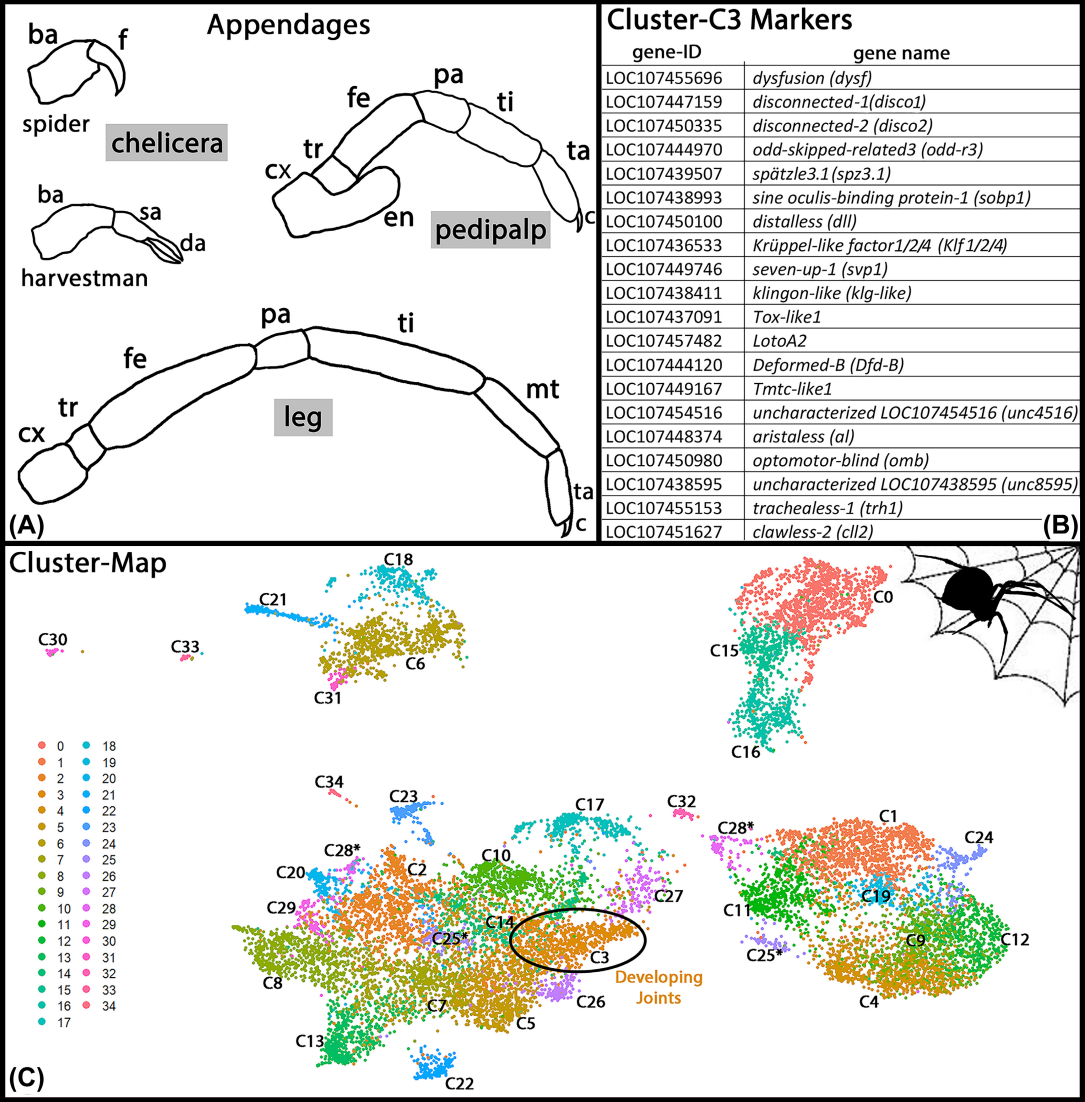
Morphology of spider and harvestman appendages, markers of Cluster‐C3, and Cluster‐map. (A) Prosomal appendages of the spider and the harvestman. Note that the chelicera of harvestmen possesses three articles, while the spider chelicera only possesses two. ba, basis; cx, coxa; c, claw; da, distal article; en, endite; f, fang; fe, femur; mt, metatarsus; pa, patella; sa, second article; ta, tarsus; ti, tibia; tr, trochanter. (B) List of markers defining cell cluster C3. (C) Cluster map. Cluster C3 is encircled. The original single‐cell sequencing data were published in Medina‐Jiménez et al.^28^

Among modern arthropods, spiders (a group of chelicerates) possess three types of serially homologous appendages on their prosoma. The most anterior pair of appendages are the chelicerae that are used to catch, hold, and sometimes also process prey, and most importantly (in spiders) are used to inject a cocktail of enzymes into their prey to kill and digest it.^7^ The second pair are the pedipalps that sometimes represent leg‐like appendages and that are used for perception, and in male spiders the tips of the pedipalps are transformed into bulbi, specialized organs that are used for sperm uptake and transfer (copulation).^8^ The third type of appendages are the four pairs of legs that are mainly used for locomotion. In different groups of spiders, their shape and especially length can vary considerably.^9^ These types of appendages have in common that they are segmented (podomerized), that is, that they are composed of separate units that are connected by (eudesmatic) joints. Additionally, they may also possess further subunits like tarsomeres (connected by adesmatic joints). The chelicerae of spiders possess two podomeres (from proximal to distal these are the base and the fang), the pedipalps possess six podomeres (from proximal to distal these are coxa [with gnathendite], trochanter, femur, patella, tibia, and tarsus). The legs possess seven podomeres (from proximal to distal these are coxa [with gnathendite], trochanter, femur, patella, tibia, metatarsus, and tarsus) (Figure 1A).^9^ Compared to the legs, the pedipalps thus lack one podomere, the metatarsus (Figure 1A). Anterior to the described prosomal appendages, there is a somewhat enigmatic structure, the labrum (upper lip). The labrum represents a special case as it is not recognized by all authors as an appendage, or at least as an appendage that is serially homologous with the other aforementioned appendages.^10^ The labrum does not possess joints. On the opisthosoma of modern spiders (Entelegynae and Haplogynae/Synspermiata), there are a number of heavily modified appendages. From anterior to posterior, these are the book lungs on the second opisthosomal segment (O2), the tracheae on O3, and two pairs of spinnerets on O4 and O5.^9^ The book lungs and the trachea form first as small limb‐like buds, but they are then internalized and function as breathing organs.^9^, ^11^ The spinnerets are flexible jointed appendages that are used to distribute the spider silk.^12^

Because of the evolutionary significance of the jointed appendages of arthropods, the evolution and development of these structures have been investigated intensively in the past, revealing quite some insights into the gene regulatory networks (GRNs) that govern joint development, at least in the main arthropod model systems, the vinegar fly *Drosophila melanogaster* and the red flour beetle *Tribolium castaneum*.^13^, ^14^, ^15^ Joint development in other groups of arthropods has not been studied as extensively and mostly relied on classical “candidate gene approaches” studying the orthologs of known *Drosophila* joint‐patterning genes.^16^, ^17^, ^18^, ^19^, ^20^, ^21^, ^22^ From these studies, we learned for example that Notch‐ and EGFR‐signaling are conserved features of joint development in the fly *Drosophila* and other insects,^14^, ^18^, ^19^, ^23^, ^24^ but also chelicerates including spiders.^16^, ^22^, ^25^, ^26^ Given the evolutionary distance between insects and spiders, the different mode(s) of appendage development, the different types of appendages and the different number of podomeres, it is likely that also the underlying GRNs (and thus involved genetic factors) may vary at least to some extent and despite the presence of conserved components. In order to overcome the general limitations of the candidate gene approach and to identify potential new genetic factors underlying the development of given morphological structures (such as the joints of arthropods) the unbiased approach of single‐cell sequencing (SCS) can be applied.^27^, ^28^

We previously published a SCS cell atlas for middle‐to‐late stage embryos of the common house spider *Parasteatoda tepidariorum* revealing several cell clusters that suggest involvement of these cells in joint development,^28^ and in a subsequent study, we provided an improved analysis of the same data set using updated versions of analytical software and applying more confined criteria for the identification of marker genes.^26^ In this improved data set, we identified a cluster that could represent cells involved in joint development, that is, Cluster‐3 (C3)^26^ (Figure 1B,C). This cell cluster is represented by 974 cells and 20 marker genes (Figure 1C and Appendix S1), and some of the genes identified in C3 are known or suggested to be involved in *Parasteatoda* joint development (i.e., *disconnected* (*disco*) (synonym *basonuclin*),^26^, ^29^ *odd‐skipped* (*odd*),^30^ *aristaless* (*al*),^31^ and *trachealess1* (*trh1*)^31^, ^32^). Others, however, represent hitherto unstudied spider genes or genes that have not been studied in the context of joint development in any arthropod species. Our data show that most marker genes of C3 are indeed expressed like typical joint‐patterning genes, that is, as rings at the places where the joints develop.

In order to shed light on the evolutionary origin of the assumed gene function in joint development, we extended our study to the harvestman *Phalangium opilio*, representing a distantly related group of chelicerates.

## 2 RESULTS

### 2.1 Determination of spider gene orthology and nomenclature

Gene orthology of the following *Parasteatoda* genes has been determined by reciprocal BLAST searches (rBLAST) (applying BLASTp) against the published sequences of the vinegar fly *Drosophila*: *dysfusion* (*dysf*) (LOC107455696), *disconnected‐1* (*disco1*) (LOC107447159), *disconnected‐2* (*disco2*) (LOC107450335), *sine oculis‐binding protein‐1* (*sobp1*) (LOC107438993), and *seven‐up‐1* (*svp1*) (LOC107449746). In these cases, we relied on rBLAST because the e‐values are supporting orthology convincingly; the reciprocal BLAST analyses of the fly genes against the spider database identified the same genes (Table 1).

**TABLE 1:** *Parasteatoda* gene orthology.

*Parasteatoda* gene	1st *Drosophila* BLAST‐hit	2nd^+^ *Drosophila* BLAST‐hit	Relevant back hits
*dysfusion* (*dysf*) LOC107455696	e‐value of 0 (*dysf*)	e‐value of 1e^−13^ (*clock*)	e‐value of 1e^−170^ (LOC107455696 = *dysf*) e‐value of 1e^−16^ (LOC107457394)
*disconnected* (*disco*) LOC107447159	e‐value of 2e^−39^ (*disco*)	e‐value of 6e^−38^ (*disco‐related*)	e‐value of 3e^−42^ (LOC107450335 = *disco2*) e‐value of 2e^−41^ (LOC107447159 = *disco*) e‐value of 2e^−04^ (LOC122269330 = *zfp271*)
*disconnected‐2* (*disco2*) LOC107450335	e‐value of 2e^−40^ (*disco*)	e‐value of 4e^−38^ (*disco‐related*)	e‐value of 3e^−42^ (LOC107450335 = *disco2*) e‐value of 2e^−41^ (LOC107447159 = *disco*) e‐value of 2e^−04^ (LOC122269330 = *zfp271*)
*sine oculis‐binding protein‐1* (*sobp1*) LOC107438993	e‐value of 7e^−43^ (*sobp1*)	e‐value of 1e^−04^ (*scm*)	e‐value of 4e^−44^ (LOC107438993 = *sobp1*) e‐value of 3e^−41^ (LOC107440400 = *sobp2*) no further hits
*seven‐up‐1* (*svp1*) LOC107449746	e‐value of 0 (*svp*)	e‐value of 9e^−68^ (*hnf4*)	e‐value of 0 (LOC107449746 = *svp1*) e‐value of 0 (LOC107457543 = *svp2*) e‐value of 6e^−80^ (LOC107454254 = rxrA)
*klingon‐like* (*klg‐like*) LOC107438411	e‐value of 1e^−58^ (*klg*)	e‐value of 9e^−56^ (*CG7166*) e‐value of 9e^−48^ (*CG34353*) e‐value of 4e^−44^ (*wrapper*)	e‐value of 2e^−58^ (LOC107438411 = *klg‐like*) e‐value of 7e^−53^ (LOC107446402)
*Tox‐like1* LOC107437091	e‐value of 3e^−15^ (*CG12104*)	e‐value of 1e^−6^ (HMGZ protein)	e‐value of 1e^−13^ (LOC107437091 = *Tox‐like1*) e‐value of 4e^−13^ (LOC107452080 = *Tox‐like2*) e‐value of 2e^−3^ (LOC107456825)
*Tmtc‐like1* LOC107449167	e‐value of 0 (*Tmtc1*)	e‐value of 2e^−180^ (*Tmtc2*)	e‐value of 0 (LOC107438659 = *Tmtc‐like2*) e‐value of 2e^−180^ (LOC107449167 = *Tmtc‐like1*) e‐value of 9e^−93^ (LOC107450269)
*unc4516* LOC107454516	e‐value of 5e^−05^ (*reph*)	No 2nd hit	e‐value of 5e^−05^ (LOC107451797 = *unc4516.2*) e‐value of 1e^−3^ (LOC107454516 = *unc4516*)
*unc8595* LOC107438595	e‐value of 1e^−17^ (*CG43896*)	e‐value of 3e^−14^ (*CG31007*) e‐value of 6e^−12^ (*CG7290*) e‐value of 1e^−11^ (*CG17826*) e‐value of 6e^−9^ (*obstructor E*)	e‐value of 4e^−21^ (LOC107438595 = *unc8595*) e‐value of 3e^−15^ (LOC107441530)

The spider gene with the identifier LOC107438411 shows most similarity to the *klingon* (*klg*) gene of *Drosophila*. Other *Drosophila* genes, however, share very similar levels of similarity, such as *CG7166*, *CG34353*, and *wrapper* (Table 1). We named this gene *klingon‐like* (*klg‐like*). The *Parasteatoda* high‐mobility group box superfamily gene with the identifier LOC107437091 is most similar to *Drosophila CG12104*, albeit with a high *p*‐value (Table 1). The rBLAST reveals most similarity to another *Parasteatoda* gene, LOC107452080, which is likely a paralog of LOC107437091. Due to the unclear phylogenetic position of LOC107437091, we simply named it *Tox‐like1*, accounting for the fact that this gene belongs to the Tox‐family of genes.^33^ The *Parasteatoda Tmtc‐like1* gene (LOC107449167) is most similar to *Tmtc1* in *Drosophila*. Given the very low e‐values for both *Drosophila Tmtc1* and *Tmtc2* and the resulting uncertainty concerning the orthology of the spider gene, we simply name it *Tmtc‐like1* (Table 1). The uncharacterized spider gene with the identifier LOC107454516 is most similar to *regulator of eph* in *Drosophila*, but with a high e‐value (Table 1). The rBLAST search shows most similarity to LOC107451797, which is likely a paralog of *unc4516*. Because of the relatively high *p*‐value, however, we decided to call this gene *unc4516* (an amalgamation of “uncharacterized” and the genes identifier (LOC107454516); please note that there is no connection to “unc” (uncoordinated) genes in the nematode worm *Caenorhabditis elegans*). Similarly, in a rBLAST search, the uncharacterized spider gene with the identifier LOC107438595 is most similar to *Drosophila CG43896* and a number of additional CG genes (*CG31007*, *CG7290*, *CG17826*), and *obstructor‐E*. Because of the very similar and high e‐values and the fact that the spider gene is similar to several genes from *Drosophila*, we simply named this gene *unc8595*.

For *odd‐skipped‐related‐3* (*odd‐r3*) (LOC107444970), we performed a phylogenetic analysis because there are several *odd*‐family genes in both insects and spiders, and because orthologs of this gene have previously been investigated in another spider, *Cupiennius salei*.^34^ Relevant for the current paper is the fact that one ortholog of the identified *Cupiennius odd‐related* genes (*odd‐r1*) has been investigated in the context of its potential role as a joint‐patterning gene.^16^ It was therefore important to identify the orthology of these genes in *Cupiennius* and *Parasteatoda*. Our phylogenetic analysis shows that the spider *odd*‐family genes cluster separately from the *odd*‐family genes of the insects *Tribolium* and *Drosophila*, and the single identified *odd* genes in the harvestman *Phalangium* (Appendix S2). We named the three *Parasteatoda* genes according to the nomenclature that was introduced by Damen et al.^34^ for *Cupiennius* (*odd‐r1* (LOC107451631), *odd‐r2* (LOC107441358), and *odd‐r3* (LOC107444970)). Note that the *Parasteatoda odd‐r3* was simply called *odd* in a previous study.^30^

We performed a phylogenetic analysis for *spätzle* (*spz*)‐family genes^35^ because there are several related genes that cannot unambiguously be separated from each other by a simple BLAST search (Appendix S3). The identified C3‐marker gene (LOC107439507) clusters with *spz3* genes from other arthropod species, and this monophyletic group forms the sister group to arthropod *spz4* genes. According to the tree, *Parasteatoda* possesses two paralogs of *spz3* (*Parasteatoda* paralog *spz3.2* [LOC107445238]).

In our phylogenetic analysis, the *Krüppel‐like factor* (*Klf*) gene (LOC107436533) does not cluster with any of the previously identified *Drosophila* or *Tribolium Klf* genes, but instead groups with *Klf1*, *Klf2*, and *Klf4* from *Homo sapiens*, two other *Klf* genes from *Parasteatoda*, and two genes from *Phalangium* ^36^, ^37^ (Appendix S4). According to the nomenclature of *Homo*, we named this spider gene *Klf1/2/4*.

Orthology of the identified LongToll gene *LotoA2* (LOC107457482) has been analyzed before, showing that it represents a paralog of *LotoA1* (see Benton et al.^38^ their Supplementary figure 3).

Paralogs of spider genes have been identified by means of sequence similarity (Table 1 and Appendices [Link], [Link]).

### 2.2 Determination of gene orthology and nomenclature in *Phalangium opilio*

For comparative reasons, we investigated orthologs of the C3 spider gene orthologs in the harvestman *Phalangium*. Single copy orthologs of *Phalangium odd*, *spz3*, and *Klf1/2/4* have been identified in our phylogenetic analyses (Appendices [Link], [Link]). As described for the spider, we relied on the unambiguous rBLAST search results for the single copy orthologs of *Phalangium dysf*, *disco*, *sobp*, and *svp* (Table 2). We identified *Phalangium* genes in a local BLAST search against the embryonic transcriptome of the harvestman using the spider C3 candidate genes as queries. We performed rBLAST searches (BLASTx) with these genes against *Drosophila* and *Parasteatoda*, showing that they are also most similar to the previously described genes in the spider (see previous chapter for further information). According to these results, we called these genes *Phalangium klg‐like*, *Tox‐like*, *Tmtc‐like*, *unc4516*, and *unc8595* (Table 2).

**TABLE 2:** *Phalangium* gene orthology.

*Phalangium* gene	1st *Drosophila* BLAST‐hit	2nd^+^ *Drosophila* BLAST‐hit	*Parasteatoda* BLAST‐hits
*dysfusion* (*dysf*)	e‐value of 0 (*dysf*)	e‐value of 6e^−12^ (*clock*)	e‐value of 0 (LOC107455696 = *dysf*)
*disconnected* (*disco*)	e‐value of 1e^−39^ (*disco*)	No 2nd hit	e‐value of 1e^−68^ (LOC107447159 = *disco*)
*sine oculis‐binding protein‐1* (*sobp1*)	e‐value of 1e^−40^ (*sobp*)	e‐value of 2e‐05 (*Sex comb on midleg*)	e‐value of 2e^−74^ (LOC107438993 = *sobp1*)
*seven‐up‐1* (*svp1*)	e‐value of 3e^−177^ (*svp*)	e‐value of 1e^−36^ (*dsf*)	e‐value of 0 (LOC107449746 = *svp1*)
*klingon‐like* (*klg‐like*)	e‐value of 3e^−45^ (*klg*)	e‐value of 6e^−40^ (*wrapper*) e‐value of 2e^−36^ (*CG7166*) e‐value of 5e^−33^ (*CG34353*)	e‐value of 8e^−33^ (LOC107438411 = *klg‐like*)
*Tox‐like1*	e‐value of 4e^−09^ (*CG12104*)	No 2nd hit	e‐value of 2e^−36^ (LOC107437091 = *Tox‐like1*)
*Tmtc‐like1*	e‐value of 6e^−52^ (*Tmtc1*)	e‐value of 4e^−35^ (*Tmtc2*)	e‐value of 6e^−145^ (LOC107438659 = *Tmtc‐like2*) e‐value of 1e^−142^ (LOC107449167 = *Tmtc‐like1*)
*unc4516*	e‐value of 1e^−2^ (*reph*)	No 2nd hit	e‐value of 2e^−20^ (LOC107454516 = *unc4516*)
*unc8595*	e‐value of 2e^−15^ (CG7248)	e‐value of 2e^−14^ (*CG43896*) e‐value of 1e^−13^ (*CG7298*)	e‐value of 5e^−91^ (LOC107438595 = *unc8595*)

### 2.3 Expression of C3 marker genes in the spider *Parasteatoda tepidariorum*

**FIGURE 2:**
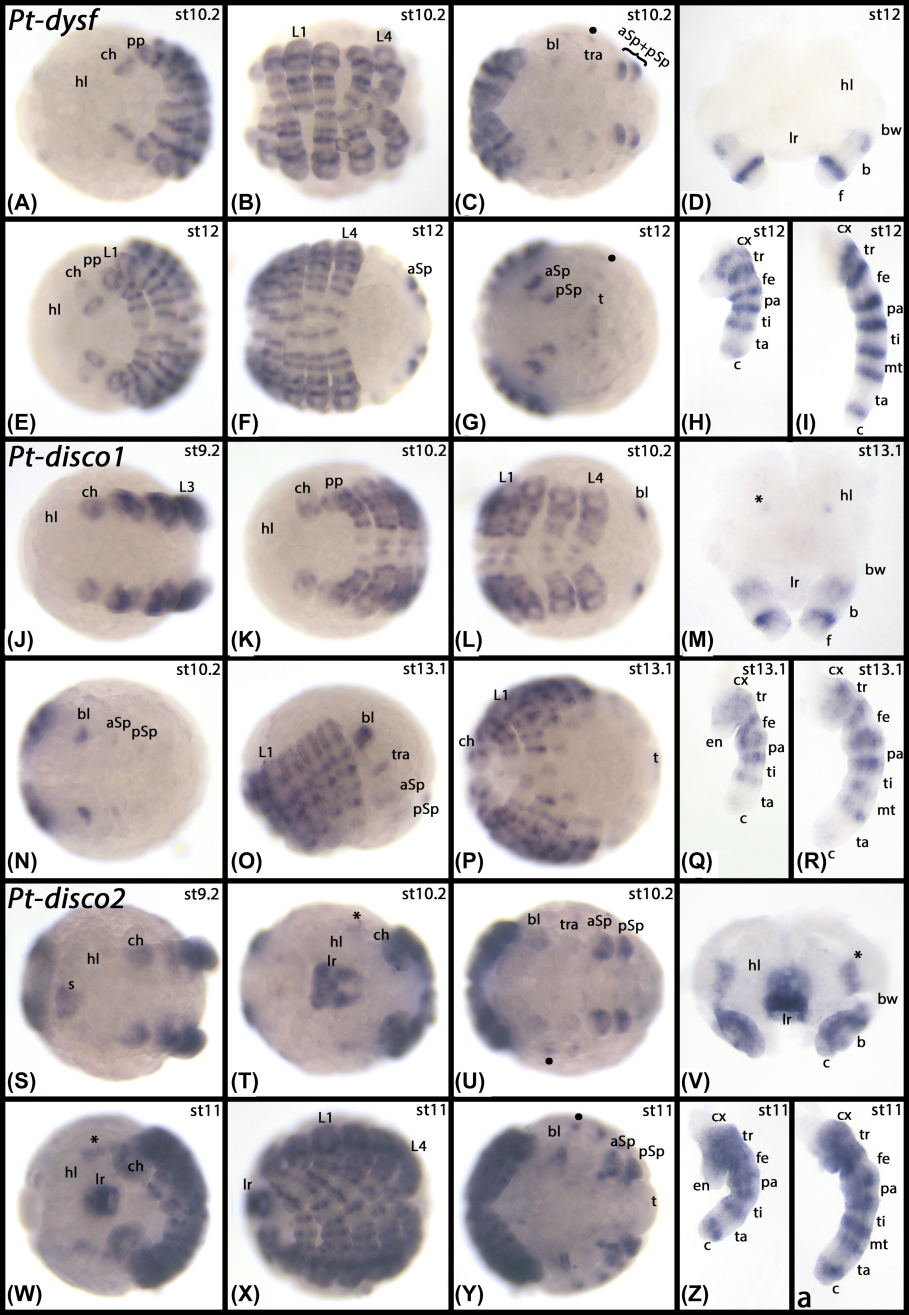
Expression of *Parasteatoda dysf*, *disco1* and *disco2*. Expression of *dysf* (A–I), *disco1* (J–R), and *disco2* (R–a). In all panels, anterior is to the left except for panels showing dissected head lobes (anterior up) and appendages (proximal up). Panels H, Q, and Z show dissected pedipalps. Panels I, R, and a show dissected legs. The filled circle in panels C, G, and Y marks faint patches of dorsal expression in the opisthosoma. The asterisks in panels M, T, V, and W mark expression in the head lobes. aSP, anterior spinneret; b, basis; bl, book lung; bw, body wall; c, claw; ch, chelicera; cx, coxa; en, endite; f, fang; fe, femur; hl, head lobe; L, leg; lr, labrum; mt, metatarsus; pa, patella; pp, pedipalp; pSp, posterior spinneret; s, stomodaeum; t, tail; ta, tarsus; ti, tibia; tr, trochanter; tra, trachea.

*Parasteatoda dysfusion* (*dysf*) is expressed in concentric rings in all developing prosomal appendages, except for the labrum (Figure 2A–I). In the opisthosoma, *dysf* is expressed in the developing book lungs, the trachea, and the two pairs of spinnerets (Figure 2C,F,G). There are two domains of expression in the chelicerae (Figure 2D), six in the pedipalps (Figure 2H), and seven in the legs (Figure 2I). These domains represent rings, except for the proximal domain in the chelicerae, which is an open circle with no ventral expression (Figure 2D). The position and number of rings suggest a function during joint development, as they correlate with the number of joints in these appendages (if the basis of the claws of legs and pedipalps are considered as joints). Apart from the described expression in the appendages, *dysf* is also expressed as segmental dots at the dorsal rim of the opisthosoma (Figure 2C,G) and in the tail region of late‐stage embryos (Figure 2G).

The two paralogs of *disconnected* (*disco*) both are markers of cluster C3. The first, *disco1*, is expressed in concentric rings in all developing prosomal appendages, except for the labrum (Figure 2J–R). The pattern of expression in the prosomal appendages is very similar compared to that of *dysf* (cf. Figure 2A–I), with the exception that the rings of *disco1* are broader than those of *dysf*, and in the pedipalps, domains corresponding to the coxa and the trochanter are not clearly defined as rings form a ubiquitous domain of expression (Figure 2Q). Compared to *dysf*, expression in the developing book lungs is stronger (Figure 2N,O). Additional expression is seen as distinct dots in the brain of late‐stage embryos (Figure 2M). The expression of *disco2* (Figure 2S‐a) is very similar to that of *disco1*, with the exceptions that expression in the brain starts earlier and is broader (Figure 2T,V,W), that there is expression in the anlage of the stomodaeum and later also the labrum (Figure 2S,T,V,W), and expression as dots at the rim of the opisthosoma (Figure 2Y). Expression in the spinnerets is stronger than that of *disco1*, but *disco2* is not expressed in the tracheal buds (Figure 2U,Y). Expression in the chelicerae is not in distinct rings (Figure 2V). As described for *disco1*, also *disco2* is expressed ubiquitously in the coxa and trochanter regions of the pedipalps (Figure 2Z). While the expression of *disco1* in the developing joint between tarsus and claw is faint, the expression of *disco2* is much stronger in this joint in both pedipalps and legs (Figure 2Z,a).

**FIGURE 3:**
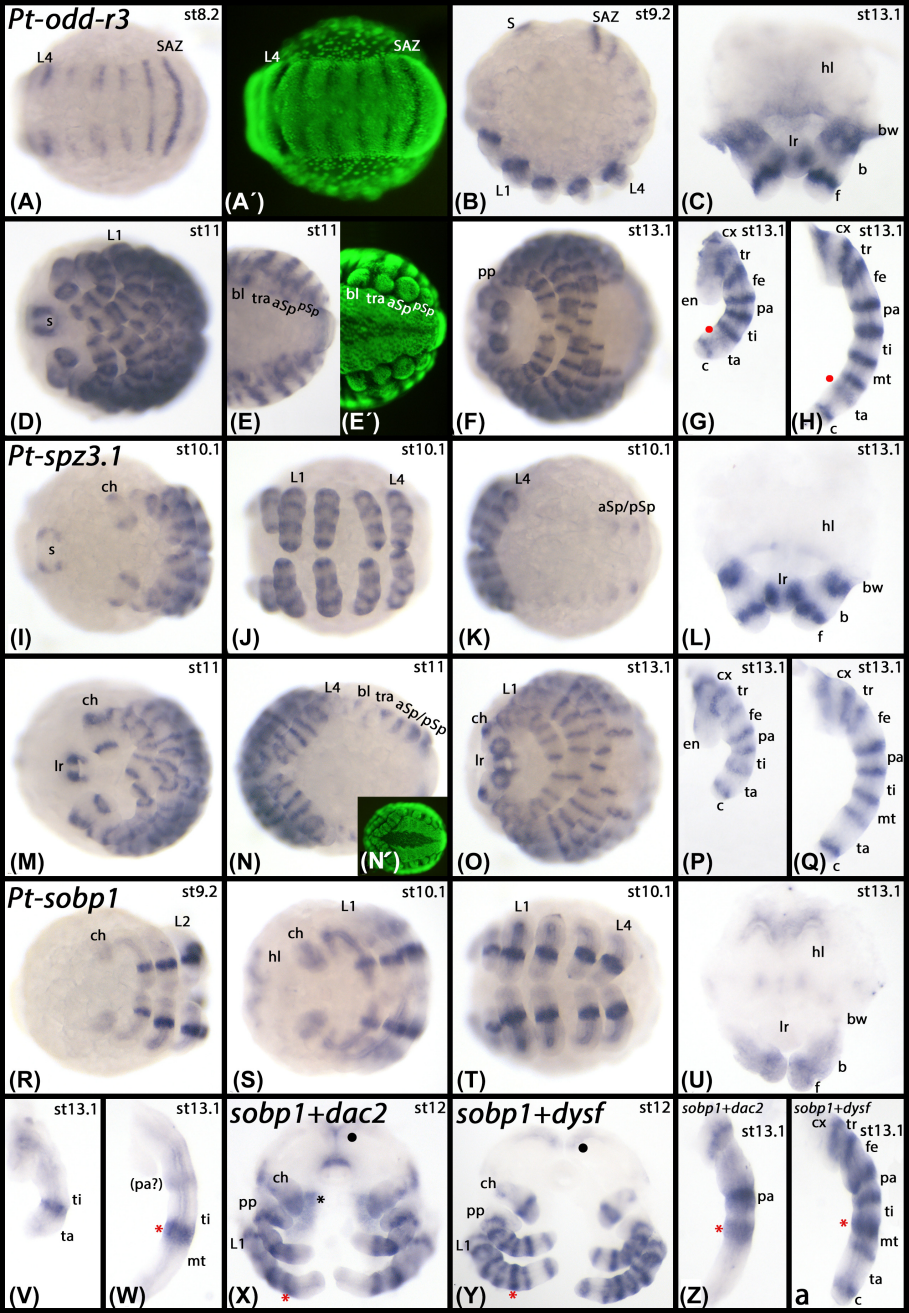
Expression of *Parasteatoda odd‐r3*, *spz3.1*, and *sobp1*. Expression of *odd‐r3* (A‐H), *spz3.1* (I‐Q), *sobp1* (R‐W), and double expression of *sobp1* and *dac2* (X, Z) and *sobp1* and *dysf* (Y, a). In all panels, anterior is to the left except for panels showing dissected head lobes (anterior up) and appendages (proximal up). Panels G, P, and V show dissected pedipalps. Panels H, Q, W, Z, and a show dissected legs. Red filled circles in panels G and H mark an additional ring of expression (see text for further information). Red asterisks in panels W and Y‐a mark expression of the strong ring of *sobp1*. The black asterisk in panel X marks expression in the central nervous system (associated with *dac2*). The filled circles in panels X and Y mark expression at the anterior rim of the head lobe (associated with *sobp1*). Panels A′ and N′ present SYBR green staining of embryos shown in panels A and N. Abbreviations: See Figure 2, and SAZ, segment addition zone.

Early during germ band development, *odd‐related‐3* (*odd‐r3*) is expressed in concentric rings in the germ disc and somewhat later as stripes in the early germ band (not shown). During posterior segment addition, transverse segmental stripes are in the anterior region of the segment addition zone (Figure 3A,B). In the maturing segments, this expression disappears, and instead, rings of expression appear in the developing prosomal appendages (Figure 3B–H). Early during development, *odd‐r3* is expressed in the anlage of the stomodaeum, and later as two lateral dots in the labrum (Figure 3B–D). The number of rings in the prosomal appendages (except for the labrum) increases during development (i.e., more rings appear over time). In the chelicerae, *odd‐r3* is expressed as two rings (Figure 3C), and in the pedipalps and legs, there are seven and eight clearly recognizable regions/rings of expression, respectively (Figure 3G,H). Within the tarsal podomere of both the developing pedipalps and the legs, however, there is an additional fainter ring of expression (Figure 3G,H [marked by red dots]). In the opisthosoma, *odd‐r3* is expressed in all limb buds, but strongest in the book lungs and as short dorsal segmental stripes (Figure 3E).

Expression of *spz3.1* is very similar to that of *odd‐3r*, with two dots in the developing labrum (Figure 3I,L,M,O), two regions/rings in the chelicerae (Figure 3I,L,M,O), seven rings in the pedipalps (Figure 3M,O,P), and eight rings in the legs (Figure 3Q). The number of rings in these appendages increases during development (cf. Figure 3J,P,Q). Unlike *odd‐3r*, however, there is no additional ring of *spz3.1* expression in the tarsal podomere of the pedipalps and the legs (Figure 3P,Q). All opisthosomal appendages also express *spz3.1* (Figure 3K,N).

The expression of *Parasteatoda sine oculis‐binding‐protein* (*sobp1*) is fundamentally different from that of the previously described C3 marker genes as it is not expressed as multiple rings in the developing appendages but instead is expressed as one very strong ring in about the center of the developing pedipalps and legs (but not so in the chelicerae) (Figure 3R–W). At later developmental stages expression appears in the interface of the basis and the fang in the chelicerae (Figure 3U) and in an additional faint ring more towards the proximal part of the legs (Figure 3W). Co‐expression of the confirmed patella‐marker *dachshund‐2* (*dac2*)^20^ and *sobp1* (Figure 3X) and the previously described *dysf* and *sobp1* (Figure 3Y) reveals that the strong *sobp1*‐domain is located distally compared to the patella (Figure 3Z [*Sobp1* marked with red asterisk]). Since the faint proximal expression of *sobp1* is not visible in this simple monochromatic double in situ, it is likely that it is covered by the stronger expression of *dac2* and thus correlated with the developing patella (Figure 3Z). Double expression with *dysf* supports the assumption that the strong domain of *sobp1* is associated with the tibia and/or the metatarsus (of legs) and tarsus (of pedipalps) (cf. Figure 3W,a). Apart from expression in the prosomal appendages, *sobp1* is also expressed anteriorly in the head lobes and as several patch‐like domains in different regions of the developing brain (Figure 3S,U).

The expression of *Parasteatoda Distal‐less* (*Dll*) has been described before.^39^ Within the domain of *Dll* expression in the appendages, expression is slightly upregulated in the region of the developing joints (Appendix S5).

**FIGURE 4:**
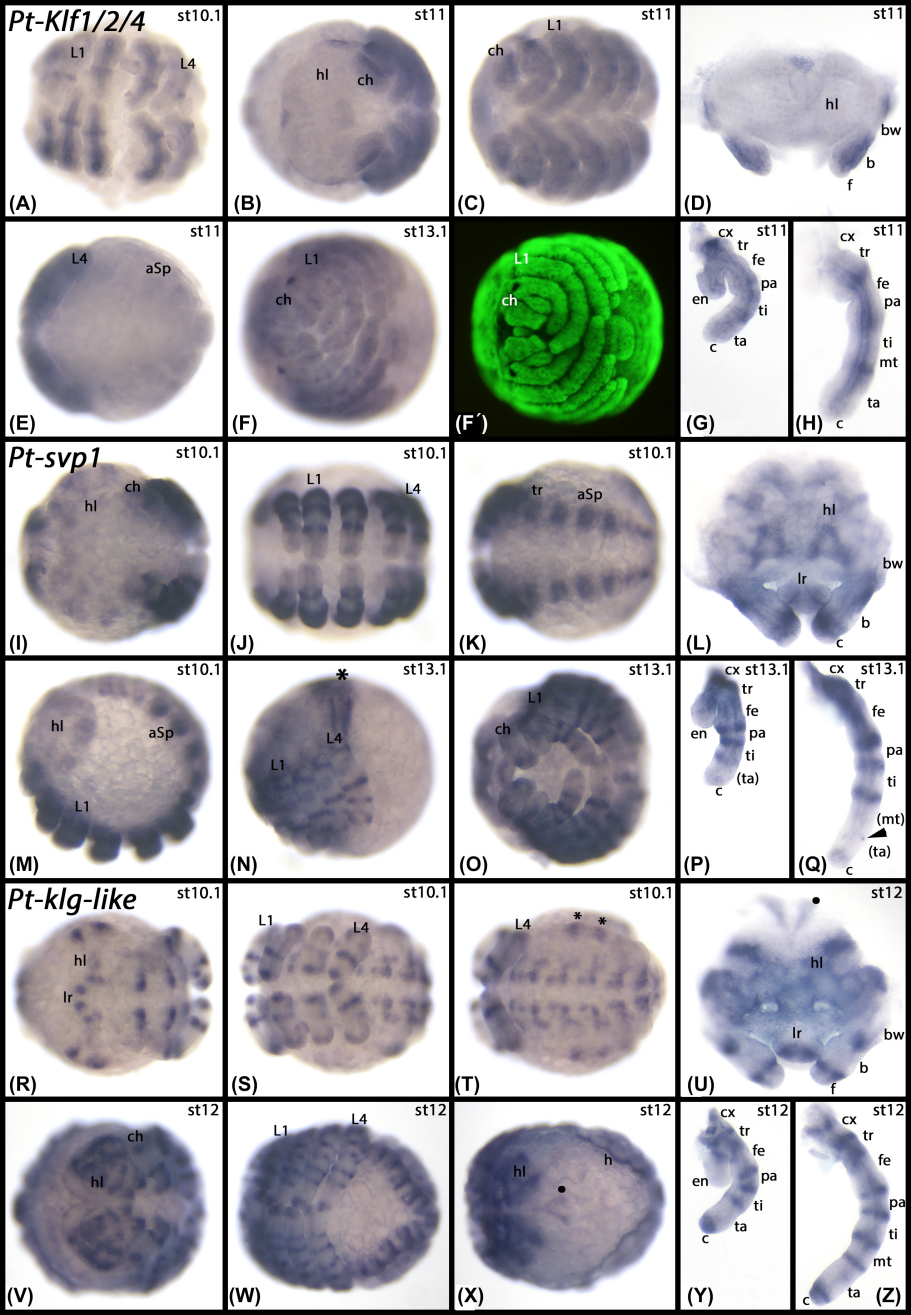
Expression of *Parasteatoda Klf1/2/4*, *svp1*, and *klg‐like*. Expression of *Klf1/2/4* (A–H), *svp1* (I–Q), and *klg‐like* (R–Z). In all panels, anterior is to the left except for panels showing dissected head lobes (anterior up) and appendages (proximal up). Panels G, P, and Y show dissected pedipalps. Panels H, Q, and Z show dissected legs. The arrowhead in panel Q points to a single dot of expression in the distal region of the leg. The asterisk in panel N marks dorsal stripes of expression. The asterisks in panel T mark patch‐like expression domains at the dorsal rim of the opisthosoma. The filled circles in panels U and X mark expression anterior to the anterior rim of the head lobes. Note that this expression may be associated with the developing heart. Panel F′ presents SYBR green staining of the embryo shown in panel F. Abbreviations: see Figure 2, and h, heart.

*Parasteatoda Krüppel‐like factor 1/2/4* (*Klf1/2/4*) is first expressed inside the developing limbs and as a single ectodermal ring (Figure 4A). Slightly later, patches of expression appear laterally in the head lobes and anteriorly in the head lobes (Figure 4B,D). Later during development, three rings of expression are visible in the legs and the pedipalps, but there are no distinct rings in the chelicerae (Figure 4C,D,G,H), and neither is there any expression in the opisthosomal limb buds (Figure 4E). At late developmental stages, the ring‐like expression transforms into patches of expression in the legs and pedipalps (Figure 4F).

The *seven‐up‐1* (*svp1*) gene is expressed in a complex pattern in the head lobes (Figure 4I,L,M). In chelicerae, pedipalps, and legs, *svp1* is expressed in several ring‐like domains, but the ring/domain that separates the tarsal podomere (in the developing legs and pedipalps) from the claw is very weak (Figure 4J,L,N–Q). In both the legs and the pedipalps, the proximal rings associated with the coxa and the trochanter are less clear than those associated with the other podomeres (Figure 4P,Q). Within the legs, a ring between the metatarsal and the tarsal podomere is missing; instead, there is a single dot of expression in the tarsal region (Figure 4Q). At late developmental stages, additional expression is seen in a broad dorsal band/stripe that separates the prosoma from the opisthosoma (Figure 4N).

*Parasteatoda klingon‐like* (*klg‐like*) is expressed in the head lobes where the brain forms and the ventral nervous system (Figure 4R–X). Additional patches of expression are at the dorsal rim of the opisthosoma (Figure 4T) and later the entire developing heart expresses *klg‐like* (Figure 4X); note the expression anterior to the head lobes that is likely associated with the heart as well (Figure 4X; marked with a filled circle) (cf. expression of the heart marker *tinman* (*tin*) in another spider^40^). *klg‐like* is also expressed in all developing appendages including the labrum and the opisthosomal limb buds (Figure 4R–W,Y,Z). First, there is expression in the tips of the developing pedipalps and legs and two rings in each of these appendages (Figure 4R–T), but at later developmental stages, there are seven rings of expression in the pedipalps and eight in the legs (Figure 4Y,Z).

**FIGURE 5:**
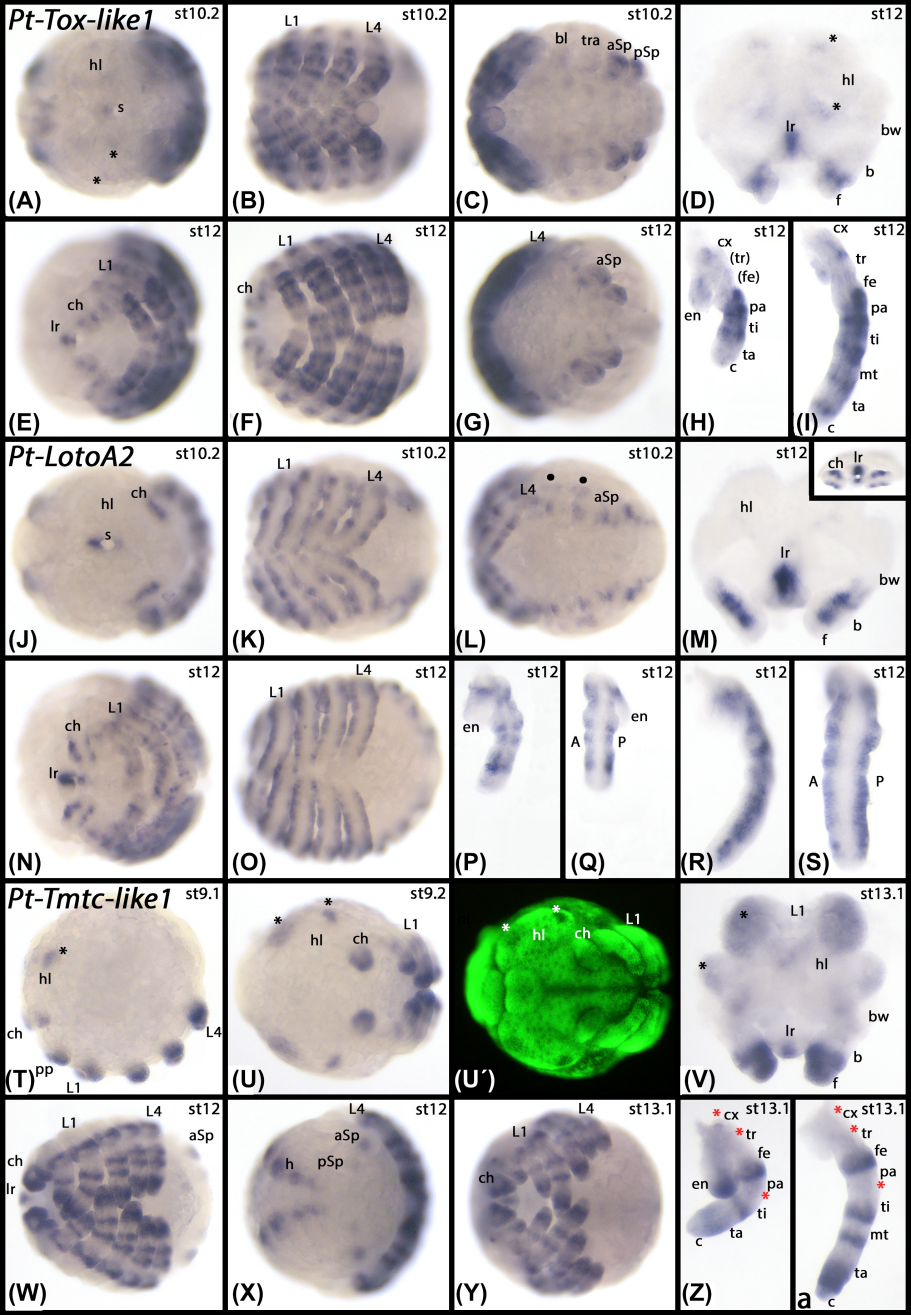
Expression of *Parasteatoda Tox‐like1*, *LotoA2*, and *Tmtc‐like1*. Expression of *Tox‐like1* (A–I), *LotoA2* (J–S), and *Tmtc‐like1* (T–a). In all panels, anterior is to the left except for panels showing dissected head lobes (anterior up) and appendages (proximal up). Panels H, P, Q, and Z show dissected pedipalps. Panels I, R, S, and a show dissected legs. Panels Q and S show top views. Asterisks in panels A, D, T, U, and V mark expression in the head lobes. Filled circles in panel L mark patch‐like domains at the dorsal rim of the opisthosoma. The insert in panel M shows a view from below on the dissected head lobe (plus chelicerae) shown in the same panel. Red asterisks in panels Z and a mark the position of missing rings. Panel U′ presents SYBR green staining of the embryo shown in panel U. Abbreviations: See Figure 2, and A, anterior; P, posterior.

*Parasteatoda Tox‐like1* is expressed in the labrum and all prosomal and opisthosomal appendages, albeit only weakly in the book lungs and the tracheae (Figure 5A–I). The domain in the labrum is central (Figure 5A,D–F). There is one distinct domain/ring in the chelicerae at the interface between the basis and the fang (Figure 5D,E). In the pedipalps there are two distinct rings in the region between the patella and the tarsus. These rings, however, are less clear than for other previously described genes of cluster C3. The area between these rings expresses *Tox‐like1* weakly (Figure 5E,F,H). In the legs, there are four distinct rings of expression associated with the region between the patella and the claw. Like in the pedipalp, there is also weaker expression in the regions between these rings (Figure 5B,E,F,I). In both developing pedipalps and legs, there is a patch of strong dorsal expression in the femur, and in the legs, there is a weaker dorsal patch at the interface between the coxa and the trochanter (Figure 5H,I). There are four weak patch‐like domains of expression in the head lobes (Figure 5A,D).

*Parasteatoda LongTollA2 (LotoA2)* is expressed in a central region of the labrum (Figure 5J,M,N) and in all other developing appendages (Figure 5J–S). Unlike all previously described C3‐cluster genes, *LotoA2* is not expressed in distinct rings but instead is expressed along the anterior and posterior margins of at least the chelicerae, the pedipalps, and the legs (Figure 5K,L,M–S; also see inlay in panel M showing a ventral view on the labrum and the chelicerae). Additional expression of *LotoA2* is in patches at the dorsal rim of the opisthosoma (Figure 5L).

The expression of *Parasteatoda Deformed‐B* (*Dfd‐B*) has been described before.^41^ Within the domain of *Dfd‐B* expression in the appendages, expression is slightly upregulated in the region of the developing joints (Appendix S5).

The *Tmtc‐like1* gene is expressed (as four patches) in the head lobes (Figure 5T–V), the spinnerets (albeit stronger in the anterior compared to the posterior spinnerets [Figure 5W,X]), and the heart (Figure 5X). Within the developing prosomal appendages, *Tmtc‐like1* is expressed as a distal patch in the labrum, two domains in the chelicerae, three rings in the pedipalps (plus distal expression in the endite), and four rings/domains in the legs (Figure 5V,Y,Z,a). Note that the rings/domains in the legs are broader than for most of the previously described C3 marker genes. A comparison with *dysf* reveals that there are no rings/domains (joint expression) separating the body wall from the coxa, the coxa from the trochanter, and the patella from the tibia in both the pedipalps and the legs (Figure 5Z,a and Appendix S5).

**FIGURE 6:**
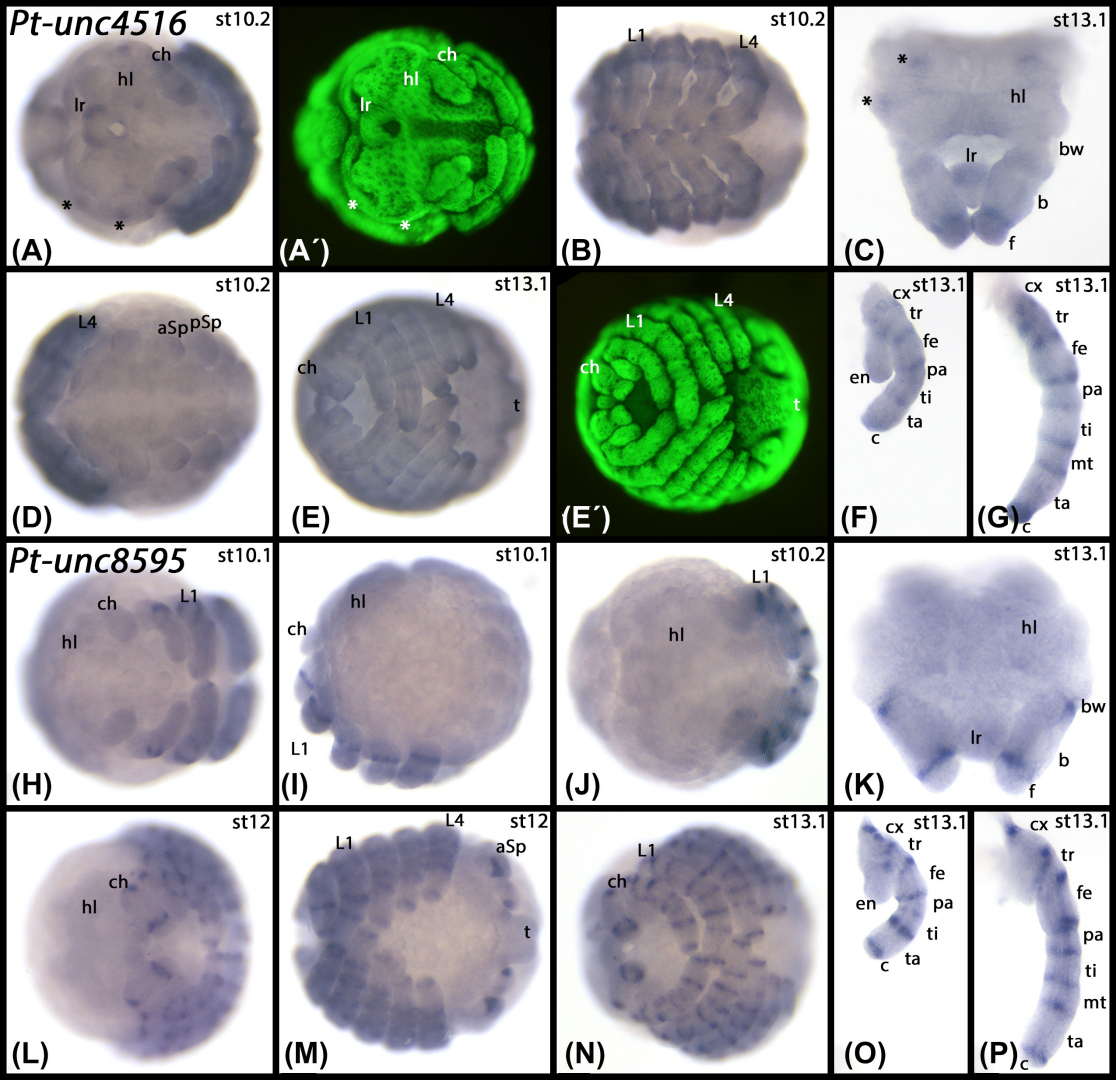
Expression of *Parasteatoda unc4516* and *unc8595*. Expression of *unc4516* (A–G) and *unc8595* (H–P). In all panels, anterior is to the left except for panels showing dissected head lobes (anterior up) and appendages (proximal up). Panels F and O show dissected pedipalps. Panels G and P show dissected legs. Asterisks in panels A and C mark expression in the head lobes. Panels A′ and E′ present SYBR green staining of the embryo shown in panels A and E. Abbreviations: See Figure 2.

*Parasteatoda unc4516* is expressed as four discrete domains in the head lobes and two domains in the labrum (Figure 6A,C). Several ring‐like domains are in the developing pedipalps and the legs (Figure 6B). Within the chelicerae, there is one discrete ring separating the fang from the basis, and a weaker ring between the basis and body wall (Figure 6A,C). The opisthosomal appendages express *unc4516* weakly (Figure 6D). At later developmental stages, there are six rings of expression in the pedipalps and seven in the legs (Figure 6E–G).

The *Parasteatoda* gene *unc8595* first is expressed as a dorsal half ring in the proximal region of the pedipalps and the legs, but not the chelicerae or the labrum (Figure 6H,I). Later during development, additional ring‐like domains appear in the pedipalps and the legs (Figure 6J), and again later expression also appears in the chelicerae between the basis and the fang and at the interface between the basis and the body wall (Figure 6K–N). In the pedipalps, there are finally seven domains of *unc8595* expression (Figure 6O). The pattern in the legs is similar to that in the pedipalps, but there are eight domains (Figure 6P). At late developmental stages, there is also expression in the anterior and the posterior spinnerets, but not in the tracheae or the book lungs (Figure 6M).

### 2.4 Expression of *Parasteatoda* C3‐cluster gene paralogs

The expression of *odd‐r1* (Appendix S6) is very similar to that of *odd‐r3* (cf. Figure 3A–G). *odd‐r3*, however, is also expressed in an additional ring within the tarsal segment of both pedipalps and legs (cf. Figure 3G,H and Appendix S6). *odd‐r2* is exclusively expressed in the pedipalps and the legs (Appendix S6), and the number of rings in these two types of appendages is different compared to *odd‐r1* and *odd‐r3* (cf. Figure 3G,H and Appendix S6).

We could not detect any specific expression of *spz3.2* (LOC107445238).

*Sobp2* is expressed in the labrum, ventrally in the chelicerae, and in the endites of the pedipalps (Appendix S7). At later developmental stages, there it is also expressed as several patches in the head lobes (Appendix S7). Note that there is no specific expression in the developing pedipalps, legs, and opisthosomal appendages.

The expression of *svp1* (Figure 4I–Q) and *svp2* (Appendix S7) is very similar. The main differences are that the pattern of *svp2* in the head lobes is more complex than that of *svp1* (cf. Figure 4L and Appendix S7). Also, *svp2* is expressed earlier and stronger in the developing labrum (cf. Figure 4I,L and Appendix S7), and dorsal expression is weaker than that of *svp1* (cf. Figure 4N and Appendix S7). Most importantly for the context of this paper, unlike *svp1*, *svp2* is also expressed in the tips of the legs and pedipalps, likely associated with claw development (Appendix S7), and in a faint ring separating the metatarsus and the tarsus in the legs (Appendix S7).

*Parasteatoda Tox‐like2* (LOC107452080) is expressed as several dot‐like domains in the head lobes and as distinct segmental patches along the ventral midline. Likely, this expression is associated with the developing nervous system of the spider (Appendix S7). In the appendages, *Tox‐like‐2* is expressed as broad domains (Appendix S7). Faint expression is also in the opisthosomal appendages (Appendix S7). Later during development, expression in the prosomal appendages refines into more distinct ring‐like domains, but they do not represent all podomeres/joints (Appendix S7).

*Parasteatoda unc4516.2* (LOC107451797) is expressed in a complex pattern in the head lobes (Appendix S7). In the appendages, *unc4516.2* is expressed as ring‐like domains that broadly correspond with the location of the developing joints (Appendix S7).

### 2.5 Expression of *Phalangium* orthologs

**FIGURE 7:**
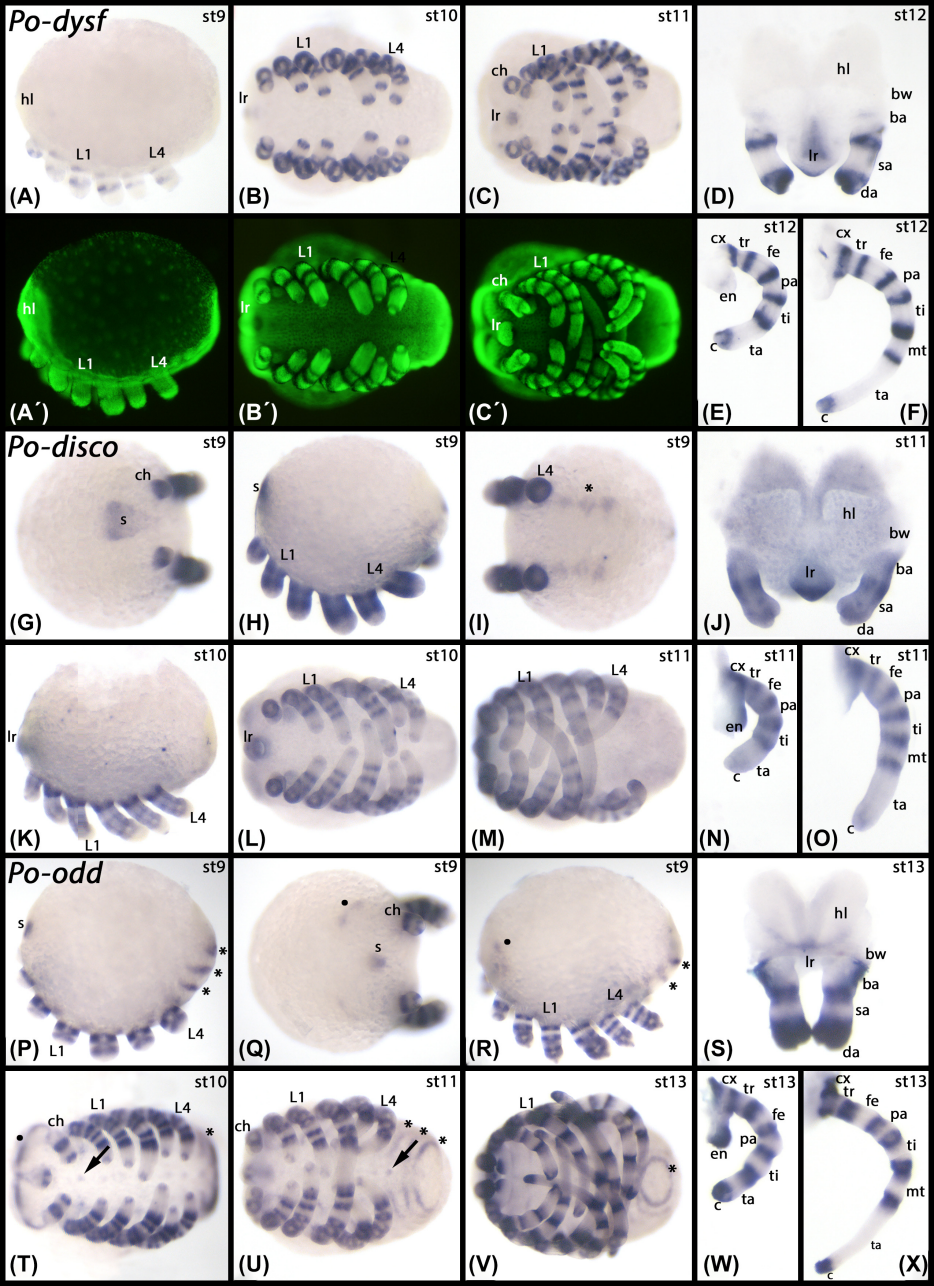
Expression of *Phalangium dysf*, *disco*, and *odd*. Expression of *dysf* (A–F), *disco* (G–O), and *odd* (P–X). In all panels, anterior is to the left except for panels showing dissected head lobes (anterior up) and appendages (proximal up). Panels E, N, and W show dissected pedipalps. Panels F, O, and X show dissected legs. The asterisk in panel I marks expression in the central nervous system. Asterisks in panels P, R, and T–V mark expression in the segment addition zone and newly formed segments. Filled circles in panels Q, R, and T mark expression in the head lobes. The arrows in panels T and U point to segmental patches of expression in the ventral midline. Panels A′–C′ present SYBR green staining of the embryo shown in panels A–C. Abbreviations: See Figure 2, and ba, basis; da, distal article; sa, second article.

*Phalangium dysf* is expressed in rings in the developing appendages (chelicerae, pedipalps, and legs) (Figure 7A–F). As these appendages mature, more rings of *dysf* appear. Finally, at late developmental stages, there are three rings/domains in the chelicerae (Figure 7D), six in the pedipalps (Figure 7E), and seven in the legs (Figure 7F). Additional expression is in the middle of the labrum (Figure 7B–D).

The harvestman *disco* gene is expressed in the anlagen of the labrum, the chelicerae, the pedipalps, and the legs (Figure 7G–O). Notably, expression in the chelicerae, pedipalps, and legs is first in a continuous proximal domain; the tips of these appendages, however, do not express *disco* or express it at a significantly lower level (Figure 7G–I). Later during development, the continuous domain in the appendages transforms into rings that increase in number (Figure 7J–O). At late developmental stages, expression of *disco* is in the tip of the labrum, in one ring in the middle of the chelicerae, six rings in the pedipalps, and seven rings in the legs (Figure 7J,N,O). The most proximal ring/domain in the pedipalps and legs is less clear than the rings (Figure 7N,O). Additional expression is in the ventral nervous system (Figure 7I).

*Phalangium odd* is expressed in rings in the developing appendages, except for the labrum in which it is expressed as two dots (Figure 7P–X). During development, additional rings of *odd* expression appear in the pedipalps and the legs (Figure 7P–X). At late developmental stages, there are three rings/domains in the chelicerae (Figure 7S), seven in the pedipalps (Figure 7W), and seven in the legs (Figure 7X). Additional expression is seen as transverse segmental stripes (at later stages, concentric rings) in/around the segment addition zone (Figure 7P,R,T–V), as segmental patches along the ventral midline (Figure 7T,U), and laterally in the head lobes (Figure 7Q,T); the latter expression disappears at later stages (cf. Figure 7S).

**FIGURE 8:**
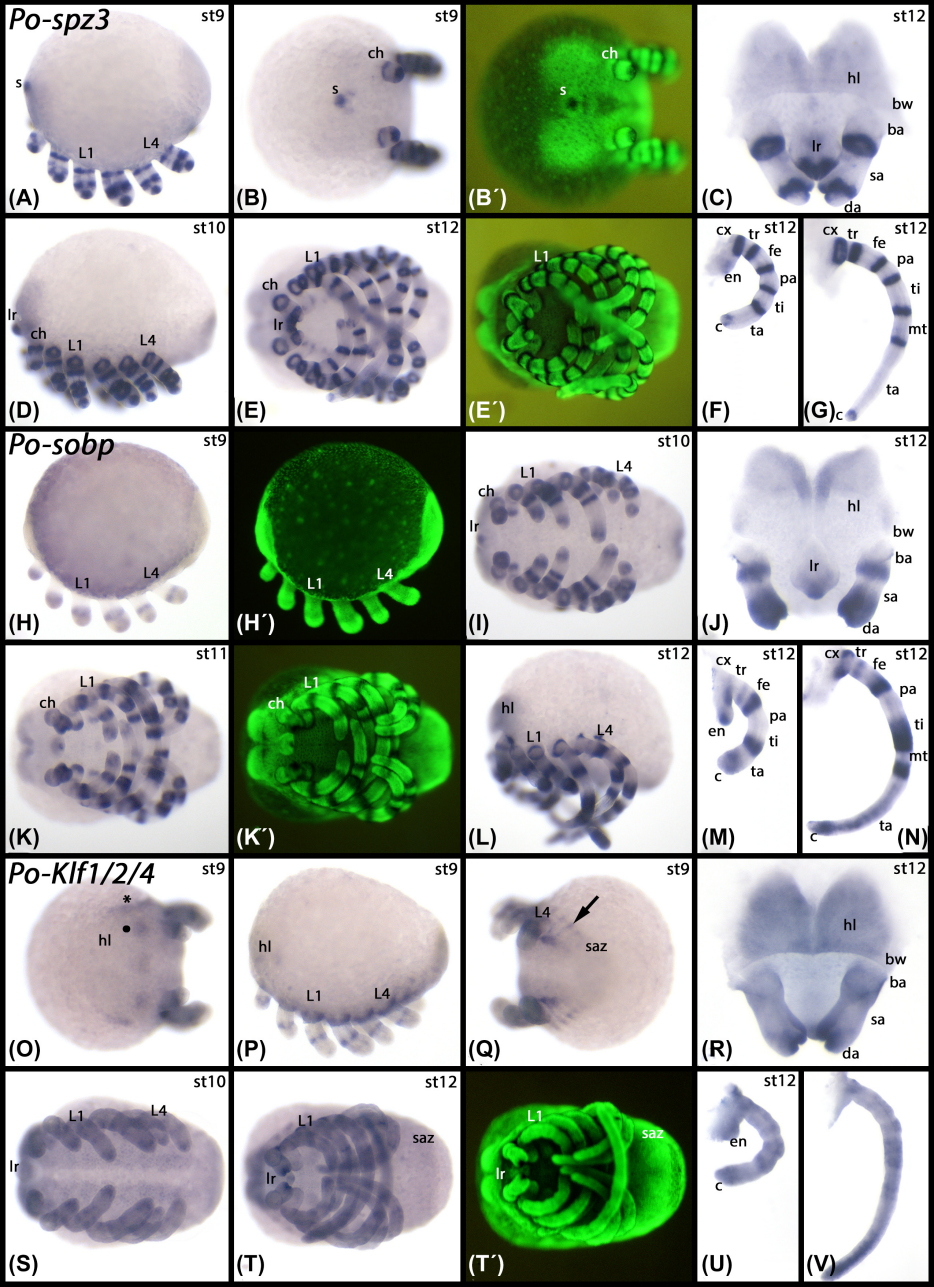
Expression of *Phalangium spz3*, *sobp*, and *Klf1/2/4*. Expression of *spz3* (A–G), *sobp* (H–N), and *Klf1/2/4* (O–V). In all panels, anterior is to the left except for panels showing dissected head lobes (anterior up) and appendages (proximal up). Panels F, M, and U show dissected pedipalps. Panels G, N, and V show dissected legs. The arrow in panel Q points to expression at the dorsal rim of the opisthosoma. The asterisk and the filled circle in panel O mark expression at the dorsal rim of the head lobe and inside the head lobe respectively. Panels B′, E′, H′, K′, and T′ present SYBR green staining of corresponding embryos. Abbreviations: See Figure 2, and ba, basis; da, distal article; sa, second article.

The harvestman *spz3* gene is exclusively expressed in the developing appendages (Figure 8A–G). In the labrum, expression is in the distal region (Figure 8C,D), but in the other appendages, there are distinct rings (Figure 8A,B,D–G). During development, the number of rings increases in both the pedipalps and the legs (Figure 8A,D–G). Finally, at late developmental stages, there are two rings in the chelicerae, six in the pedipalps, and seven in the legs (Figure 8C,F,G).

*Phalangium sobp* is expressed in all appendages (Figure 8H–N). In the labrum, expression is distal (Figure 8J,K). In the chelicerae, the pedipalps, and the legs, *sobp* is expressed in rings. The number of rings increases during development, at least in pedipalps and legs. At late developmental stages, there are two rings/domains in the chelicerae (Figure 8J); in the pedipalps, there are five rings, and six in the legs (Figure 8M,N). There is additional expression anterior in the head lobes (Figure 8J,K). Compared to most other ring‐like domains in the appendages here described, the domains of *sobp* are broader and resemble the domains of limb gap genes.

*Phalangium Klf1/2/4* is expressed laterally in the head lobes, as two patches in the head lobes, and as dorsal stripes in the opisthosoma (Figure 8O–Q). Additionally, there are one or two rings of expression in the chelicerae, pedipalps, and legs (Figure 8O–Q). Later during development, additional ring‐like domains appear, except for the chelicerae (Figure 8R–V); note that these rings are less distinct as described for other C3 marker genes and vary in their proximal to distal extension (Figure 8U,V). Additional expression is in the tip of the labrum (Figure 8R,T).

**FIGURE 9:**
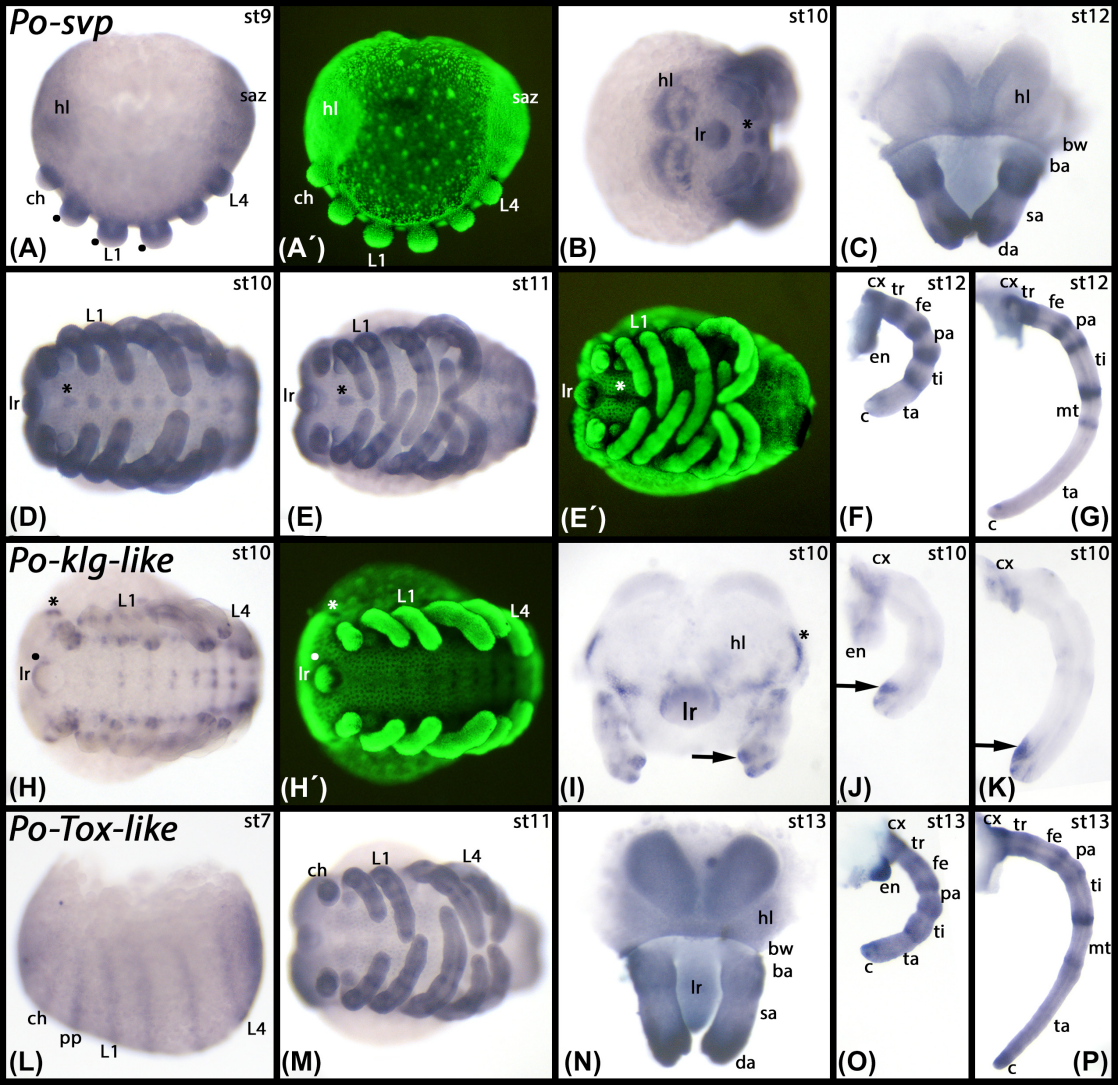
Expression of *Phalangium svp*, *klg‐like*, and *Tox‐like*. Expression of *svp* (A–G), *klg‐like* (H–K), and *Tox‐like* (L–P). In all panels, anterior is to the left except for panels showing dissected head lobes (anterior up) and appendages (proximal up). Panels F, J, and O show dissected pedipalps. Panels G, K, and P show dissected legs. Filled circles in panel A mark tips of appendages that are free from expression. Asterisks in panels B, D, and E mark expression along the ventral midline. The asterisks and the filled circle in panels H and I mark lateral expression in the head lobes and expression in the labrum, respectively. The arrows in panels I–K mark expression at the distal tip of the appendages. Panels A′, E′, and H′ present SYBR green staining of corresponding embryos. Abbreviations: See Figure 2, and ba, basis; da, distal article; sa, second article.

The single harvestman *seven‐up* gene (*svp*) is first expressed ubiquitously in the developing limb buds, albeit weaker in their distal regions (Figure 9A). Later during development, distinct ring‐like domains form (Figure 9B–G). At late developmental stages, there are two rings/domains in the chelicerae (Figure 9C), six in the pedipalps (Figure 9F), and seven in the legs (Figure 9G). Additional expression of *svp* is in the tip of the labrum (Figure 9B,D; note that this expression is covered by the chelicerae in panel C) and as segmental patches along the ventral midline (Figure 9B,D,E).

*Phalangium klg‐like* is expressed in the dorsal region of the developing labrum, laterally in the head lobes, along the ventral midline (as two dots per segment), and chelicerae, pedipalps, and legs (Figure 9H–K). Expression in the latter three types of appendages, however, is not in rings or ring‐like domains but instead is expressed predominantly as distinct distal patches and a salt‐and‐pepper‐like proximal domain (Figure 9I–K).

Already early during development, *Phalangium Tox‐like* is expressed as transverse segmental stripes in the prosoma (Figure 9L). This expression represents inter alia the primordia of the prosomal appendages. Later, distinct rings of expression appear in the outgrowing appendages (Figure 9M–P), and at late developmental stages there are two domains in the chelicerae, six in the pedipalps, and seven in the legs, suggesting a general function in joint development (Figure 9N–P).

**FIGURE 10:**
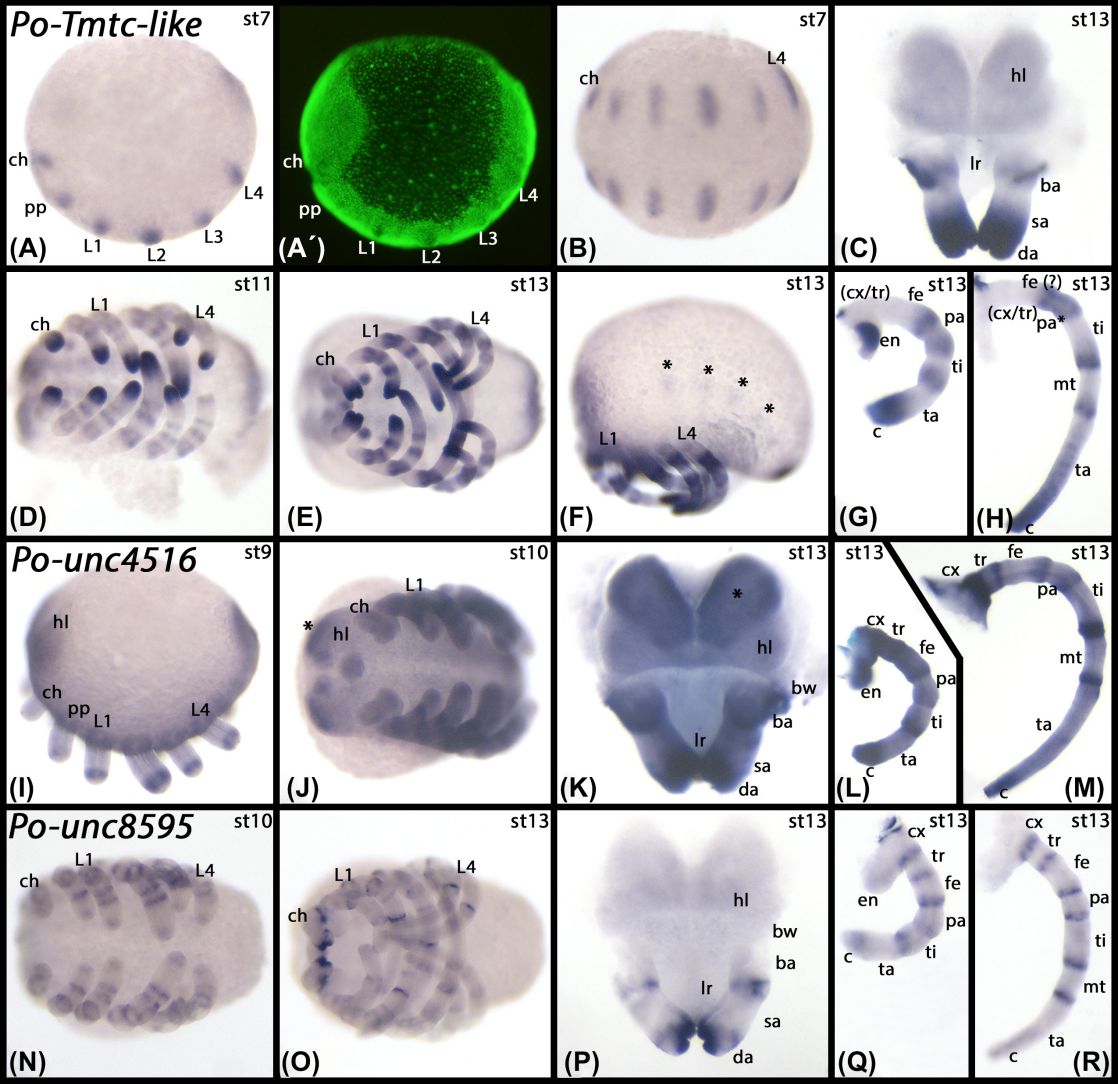
Expression of *Phalangium Tmtc‐like*, *unc4516*, and *unc8595*. Expression of *Tmtc‐like* (A–H), *unc4516* (I–M), and *unc8595* (N–R). In all panels, anterior is to the left except for panels showing dissected head lobes (anterior up) and appendages (proximal up). Panels G, L, and Q show dissected pedipalps. Panels H, M, and R show dissected legs. Asterisks in panel F mark expression along the dorsal rim of the opisthosoma. Asterisks in panels J and K mark expression in the developing brain. Panel A′ represents SYBR green staining of the embryo shown in panel A. Abbreviations: See Figure 2, and ba, basis; da, distal article; sa, second article.

*Phalangium Tmtc‐like* is expressed in the primordia of the prosomal appendages (Figure 10A,B). Later during development, as the appendages grow out, rings of expression appear (Figure 10C–H). The strongest expression is seen in the tips of the appendages except for the labrum (Figure 10C–E,G,H). The rings/domains in the developing appendages, however, do not exactly match the number and position of the joints. Although there are two domains in the chelicerae, the distal one is broad and covers the complete distal region of the chelicerae (Figure 10C–E). In the pedipalps, there are only four distal domains/rings of expression that could be associated with joint development. Domains between the body wall and the coxa, and the coxa and the trochanter, however, are clearly missing (Figure 10G), and this situation is the same in the legs (Figure 10H). In addition, the domain covering part of the femur and the patella does not suggest a distinct function in patterning the corresponding joint (Figure 10H, marked with a question mark and an asterisk). Additional expression of *Tmtc‐like* is in distinct patches at the dorsal rim of the germ band where the heart will form (Figure 10F).

When the prosomal appendages begin to grow out, *Phalangium unc4516* is first expressed in a single ring in the pedipalps and the legs (Figure 10I). Later, additional rings form that finally correspond in place and number with the developing joints (Figure 10J–M). In the pedipalps, the domains separating the body wall from the coxa and the coxa from the trochanter are much less clear than in the legs (Figure 10L,M). Additional expression of *un4516* is in the developing brain (Figure 10J,K).

The harvestman ortholog of *unc8595* is expressed in rings in the appendages (Figure 10N–R). During development, additional rings appear along the AP axis of the appendages. Finally, there are three distinct domains in the chelicerae, six in the pedipalps, and seven in the legs (Figure 10P–R).

## 3 DISCUSSION

### 3.1 Cluster‐C3 (C3): cells involved in appendage patterning and joint‐development

C3 is characterized by cells that differentially express genes known to be involved in appendage development in *Parasteatoda* (*aristaless* (*al*), *clawless2* (*cll2*), and *trachealess*1 (*trh1*),^31^ *clawless2* (*cll2*))^42^ (*odd‐skipped* (*odd*) (= *odd‐r3*)),^30^ (*disconnected* (*disco*) (= *disco1*)^28^). One specific feature of these genes is their expression in ring‐like domains along the proximal‐distal (PD) axis of the chelicerae, the pedipalps, and the legs, suggesting a role in joint development. Some of the marker genes of C3, such as *Distal‐less* (*Dll*), *Deformed‐B* (*Dfd‐B*), and *optomotor‐blind* (*omb*) have also been analyzed before, but there was no reported expression in rings along the PD axis of the appendages, although these genes are all expressed in the appendages ((*omb*),^44^ (*Dll*),^39^ (*Dfd‐B*)^41^).

This initial literature analysis prompted us to speculate that C3‐marker genes could be involved in joint‐development or at least appendage patterning in general. As our subsequent comprehensive in situ hybridization analysis of all C3 marker genes showed, these genes are indeed expressed in patterns that are in line with this hypothesis (Figures 2, 3, 4, 5, 6, Table 3). Most interestingly, the top markers of this cluster (i.e., *dysf*, *disco1*, *disco2*, *odd‐r3*, and *spz3.1*) indeed all are expressed in a pattern that suggests a general involvement in joint development as they are expressed in all developing joints (Figures 2 and 3). But even some of the “lesser” markers of C3 are expressed in rings in all developing joints (e.g., *klg‐like*, *unc4516*, and *unc8595*) (Figures 4 and 6), while others are only expressed in certain joints (e.g., *sobp1*) (Figure 3, Table 3). The latter could be explained by the fact that many spider genes are duplicated, possibly as a result of a whole genome duplication in the lineage leading to Arachnopulmonata.^41^ The possibility exists that duplicated genes undergo sub‐functionalization and are then expressed in (combined) patterns that resemble that of the original founder gene^26^, ^42^, ^43^ (critically reviewed in Sharma^45^). It is thus possible that some of the C3 marker gene paralogs also play a role in joint development or complement the “joint‐pattern” of C3 markers that are not expressed in all developing joints such as *sobp1*, *svp1*, *Tox‐like1*, or *Tmtc‐like1*. We address this in the following chapter.

**TABLE 3:** Summarized function and/or expression of investigated C3 marker genes in *Drosophila*, *Parasteatoda*, and *Phalangium* joint development.

Gene	*Drosophila* (function in joints)	*Parasteatoda* (expression in joints)	*Phalangium* (expression in joints)
***dysfusion* (*dysf*)**	Tarsal	All	All
***disconnected‐1* (*disco1*)**	Distal	All (except claw)	All
***disconnected‐2* (*disco2*)**	n.a.	All (complementing *disco1*)	n.a.
***odd‐skipped‐related3 (odd‐r3)***	All^a^	All	All
*odd‐skipped‐related2 (odd‐r2)*	(see above)	Subset	n.a.
*odd‐skipped‐related1 (odd‐r1)*	(see above)	All	n.a.
***spätzle3.1 (spz3.1)***	None	All	All
*spätzle3.2 (spz3.2)*	n.a.	None	n.a.
***sine oculis‐binding protein‐1* (*sobp1*)**	None	Subset	All
*sine oculis‐binding protein‐2* (*sobp2*)	n.a.	None	n.a.
***Krüppel‐like factor‐like1/2/4 (Klf‐1/2/4)***	None	Subset	Subset
***seven‐up1 (svp1)***	None	Subset	All
*seven‐up2 (svp2)*	n.a.	Subset (complementing *svp1*)	n.a.
***klingon‐like* (*klg‐like*)**	None	All	None
***Tox‐like1***	None	Subset	All
*Tox‐like2*	n.a.	Subset (supplementing *Tox‐like1*)	n.a.
***Tmtc‐like1***	None	Subset	Subset
*Tmtc‐like2*	n.a.	Ubiquitous expression (not informative)	n.a.
***unc4516***	None	All	All
*unc4516.2*	n.a.	All	n.a.
***unc8595***	None	All	All

*Note*: None, no function/expression in joints, or no data available; n.a., not applicable (no paralogs). Genes in bold are markers of cluster C3.

^a^ General function of *odd*‐family genes in *Drosophila* joint development.

### 3.2 Are C3 paralogs involved in appendage patterning and joint development?

The *odd‐r1* gene is expressed in almost identical patterns as *odd‐r3* (the C3 marker gene) but the extra ring within the tarsal segment is not present, and the *odd‐r2* gene is only expressed in a subset of joints (cf. Figure 3 and Appendix S6). *odd‐r1* and *odd‐r3* could thus have partially redundant functions, and the specific function of *odd‐r2* (if any) remains unclear.

The *sobp2* gene is not expressed like that of a joint‐patterning gene, revealing that it does not contribute to the development of the joints or even substitute for the “missing” joint domains of *sobp1* (Appendix S7). Consequently, *sobp* is not a general joint marker in the spider.

*Parasteatoda svp2* is expressed in the joints, including (weakly) the joints between the metatarsus and the tarsus and (strongly) the tarsus and the claw (Appendix S7), the two domains that are missing or less prominent for *svp1* (cf. Figure 4Q and Appendix S7). Together, *svp1* and *svp2* thus cover expression in all joints, suggesting that *svp* could generally be involved in the development of joints in the spider.

Similarly, *Parasteatoda Tox‐like2* is expressed in rings in the proximal region of the prosomal appendages, the regions in which *Tox‐like1* is not expressed (cf. Figure 5 and Appendix S7). These two paralogs could thus represent a case of sub‐functionalization and consequently, spider *Tox‐like* genes could be involved in the development of the joints.

The ubiquitous expression of *Tmtc‐like2* (LOC107438659) does not allow us to draw any conclusions on potential sub‐functionalization and/or a function in joint development.

The expression of *unc4516.2* in the regions where the joints form (albeit in a more diffuse patterns) may suggest a function during joint development as well.

### 3.3 Comparative data from the harvestman and other arthropods

Once we identified many of the C3 cluster genes (and some of their paralogs) as potential factors of joint development in the spider, we extended our research to the harvestman *Phalangium* in order to gain deeper insights into the evolutionary history of C3 expression patterns. Our data show that most of the harvestman orthologs of *Parasteatoda* C3 genes are indeed expressed in rings in the developing appendages of the harvestman, suggesting that their function is conserved in both species. Although this does not cover Chelicerata as a whole, it at least shows that the expression/function extends through Arachnopulmonata. The data also show that the number of joints and rings of expression correlates perfectly for *dysf*, *disco*, *odd, spz3, svp, Tox‐like, unc4516*, and *unc8595* in both the spider and the harvestman, further substantiating the assumption that these genes are indeed involved in joint development (Figures 7, 8, 9, Table 3). In the following section, we summarize gene‐by‐gene available data from other arthropods and correlate our new data with these previously published data, especially in the context of appendage‐ and joint‐development.

In *Drosophila*, *dysf* is expressed in the tarsal “joints”, but not the regular podomere joints, and only the tarsal joints fail to develop in the mutant background.^46^, ^47^ Our data suggest that this either represents a derived pattern because in the chelicerates, *dysf* is expressed in all joints, or that the pattern in the chelicerates represents a derived feature of development. Future research will be needed to address this question as there is no data on expression and/or function of *dysf* in any other hitherto studied arthropod.

In *Drosophila*, *disco* is expressed in most of the leg imaginal discs, including the most distal region, and is dependent on the activity of *Distal‐less* (another marker of the C3 cluster).^48^ Expression in the tip is different from the situation in the investigated chelicerates, the beetle *Tribolium*, and the myriapod *Glomeris*, in which the very tips of the appendages do not express *disco* equally strongly.^49^, ^50^ In both *Drosophila* and *Tribolium*, *disco* genes are upregulated in rings within their overall expression domain,^49^ and overexpression of *disco* leads to the lack or fusion of some tarsal sub‐segments.^49^, ^51^ The conserved patterns found in chelicerates and insects suggest that aspects of *disco* function are likely conserved in Arthropoda as a whole and that this function is correlated with the development of the joints. In this context, it is interesting to note that *Dll* is also expressed in several rings in chelicerates (but apparently not in other groups of arthropods)^52^ (Appendix S5). Nevertheless, depletion of *Dll* leads to distal joint defects in other arthropods, such as the beetle *Tribolium* and the true bug *Oncopeltus*.^53^, ^54^ A conserved interaction of Dll and Disco is thus likely. Further research will be needed to reveal if arthropod *disco* genes could be more generally involved in joint development, as is the case in *Drosophila*.

*Drosophila odd‐*family genes are generally involved in joint development.^55^ In the beetle *Tribolium*, the expression patterns of *odd*‐related genes in joint‐rings are conserved, and knock‐down of (at least) some members of this family leads to truncated appendages that lack some of the joints.^13^, ^56^ In *Oncopeltus*, at least one of the *odd*‐family genes is expressed in rings in the developing appendages.^57^ In *Glomeris*, the single identified *odd* gene is expressed in (at least) one ring in the developing appendages; note that the appendages grow relatively late in this species, possibly explaining the paucity of rings in the published embryos.^58^ Most importantly, an *odd*‐related gene (*odd‐r1*) has previously been identified as a joint marker in the spider *C. salei*. The function of *odd‐r1* in this spider, however, could not be revealed,^16^ likely because of the presence of other *odd‐r* genes with redundant function(s). Nevertheless, it is thus not surprising to find an *odd*‐related gene (*odd‐r3*) among the genes that characterize the C3 cluster. The spider and harvestman data (together with the previously published data) strongly suggest that *odd*‐related genes indeed represent conserved key factors of arthropod joint development.

Finding that *spz3.1* is expressed in all joints in the spider and the harvestman, however, was surprising because to our knowledge there are no published data that would suggest a role of this gene in appendage development in *Drosophila* or any other species. Instead, in *Drosophila*, *spz3* is involved in glial morphogenesis and neuronal survival and function.^59^ Data on other arthropods are not available, and it is thus currently not possible to state what the evolutionary origin of the joint‐related pattern is that we detected in the spider and the harvestman, a question that can only be addressed by the investigation of *spz3* genes in representatives of all main groups of arthropods, such as myriapods and crustaceans, but also other groups of insects.

The *sobp* gene is not expressed in all joints in the spider and the harvestman, and the pattern in both species is different (Figures 3 and 8). This shows that *sobp* is not a bona fide joint marker. Indeed, the rings/domains of *sobp* are broader than those of *odd* and other likely joint markers, suggesting that the function is not restricted to the place where the joints form. Functional data on these genes are needed to further investigate their potential function during appendage development. In *Drosophila*, *sobp* interacts with the conserved eye‐patterning gene *sine oculis* (*so*),^60^ but there is no information about a potential role in appendage patterning or joint development, and there are no other comparative data on the expression or function of *sobp* in any other arthropod species. Therefore, neither function nor evolutionary origin of the patterns seen in the developing appendages of the spider and the harvestman can be extrapolated based on the current stand of information.

At late developmental stages, *Parasteatoda Dll* is upregulated in rings in the developing appendages (Appendix S5) and this pattern is conserved in other spiders such as the tarantula *Acanthoscurria geniculata* and the cellar spider *Pholcus phalangioides*, indicating a potential role in joint development^39^, ^61^ (their supplementary figure 5). Also, in the harvestman *Phalangium*, *Dll* is expressed in rings that may be correlated with the development of the joints.^62^ In *Drosophila*, *Dll* and *Notch* are involved in the regulation of genes that control distal joint development,^14^ and also in *Tribolium*, *Dll* phenotypes cause fusion of the distal tarsi.^63^ The involvement of *Dll* in tarsal development thus appears to be conserved at least in higher insects. This raises the question of whether the ring pattern seen for *Dll* in chelicerates could indicate a more general involvement in joint development in this group of arthropods. Available functional data in different groups of chelicerates, including spiders, mites, and harvestmen, however, suggest a conserved role of *Dll* as an appendage gap gene, as knockdown always results in a drastic truncation of the appendages that express *Dll*, rather than specific defects in joint development.^62^, ^64^, ^65^ These data, however, have to be seen in the context of dose‐dependent phenotypes. Even in *Drosophila*, strong phenotypes cause a leg‐gap gene‐like phenotype, while partial loss of function can lead to milder defects only concerning the developing tarsi.^66^, ^67^ Likewise, the reported *Dll*‐RNAi phenotypes in chelicerates may represent strong phenotypes covering weaker phenotypes (that could be associated with the ring‐like expression patterns of *Dll* in these species).

The harvestman *Klf1/2/4* data show that there is no general association with the joints corroborating the spider data and suggesting that *Klf1/2/4* is not involved in the development of all joints. To the best of our knowledge, none of the *Drosophila* genes that are related to the chelicerate *Klf1/2/4* genes are involved in the development of the appendages, and there are no other comparative data from any other group of arthropods.

The harvestman and the spider *svp* gene(s) are expressed in all joints, suggesting a conserved function in at least the lineage leading to spiders and harvestmen. In *Drosophila*, *svp* is involved in the development of the nervous system and the heart, and there are no data suggesting a role in appendage development.^68^, ^69^, ^70^ Our data thus provide the first evidence that *svp* genes could be involved in arthropod joint development, at least in chelicerates.

Interestingly, the harvestman *klg‐like* gene is not expressed like a joint patterning gene although this is the case in the spider. This either suggests that the joint‐patterning expression pattern of *klg‐like* evolved in the lineage leading to *Parasteatoda*, or that it was lost in the harvestman. Further research is needed to unveil the evolutionary origin of this pattern. Comparison of the *klg‐like* gene, however, is complex because there is no clear ortholog in *Drosophila*; instead, the spider *klg‐like* gene is equally related to a number of different *Drosophila* genes such as *CG7166*, *CG34353*, *wrapper*, and indeed also *klg* (discussed above). Neither of these genes, however, has been described as an appendage patterning factor. *Drosophila klg* is involved in nervous system development,^71^ and the *wrapper* gene is expressed in midline glia cells.^72^ There is no information about *CG7166* and *CG34353*.

Expression of *Parasteatoda Tox‐like1* and *Tox‐like2* suggests a role in joint development, although the patterns are less restricted to the exact position where the joints form than seen for other C3 marker genes. The harvestman data support this assumption as its Tox‐like gene is expressed at the interface of every podomere in the developing chelicerae, pedipalps, and legs (Figure 9M–P). The most closely related *Drosophila* gene, *CG12104*, is expressed in the proventriculus, and there is no information about a potential expression or function in the appendages (BDGP in situ database homepage; https://insitu.fruitfly.org/cgi‐bin/ex/report.pl?ftype=3&ftext=LP01188; 11th of June 2024). To our knowledge, there is no information on Tox‐like function or expression in any other arthropod species.

The *Parasteatoda LotoA2* gene has been identified in a previous study, but its expression has not been reported.^38^ Among the newly investigated C3‐cluster genes, *Parasteatoda LotoA2* is the only gene that is not expressed in any rings, but instead it is expressed along the anterior and the posterior margin of the appendages (Figure 5). To the best of our knowledge, there is no information about a possible function of long toll genes in *Drosophila* appendage development. This pattern is not conserved in other groups of arthropods and onychophorans.^38^, ^73^ The function of *LotoA2* in *Parasteatoda* appendage development thus remains unclear, and we did not further investigate the evolutionary origin of this pattern.

We have shown that the Hox gene *Dfd‐B* is upregulated in rings in the legs of *Parasteatoda* (the body region that expresses *Dfd‐B*) at late developmental stages (Appendix S5), and this pattern has also been observed in other spiders such as *Cupiennius* ^74^ and *Pholcus*,^75^ and the harvestman *Phalangium*.^76^ *Dfd* expression has also been investigated in a scorpion and a horseshoe crab, but the published data are not fully conclusive with respect to possible expression in rings.^77^ To our knowledge, there is no data on a possible function of *Dfd* in joint development in these species or indeed any other arthropod. Interestingly, however, knockdown of *Dfd* in *Phalangium* leads to homeotic transformations, but the joints of these transformed appendages appear to develop normally, implying that *Dfd* may not be involved in joint development.^25^

The spider *Tmtc‐like1* gene is expressed in some joint‐regions, but in both pedipalps and legs, expression is missing at the interface between body wall and coxa, coxa and trochanter, and femur and patella (Figure 5Z,a). Since the expression of its paralog is ubiquitous, we cannot receive any additional information from it, but interestingly, the pattern in the harvestman *Phalangium* is conserved with respect to the spider *Tmtc‐like1* gene. Like in the spider, corresponding joint‐domains are lacking in the harvestman, strongly suggesting a conserved function and that *Tmtc‐like1* does not generally contribute to the patterning and development of all joints. The relatively large (PD‐extension) domain of *Tmtc‐like1* in the spider suggests another role but joint‐specification, and this pattern is also seen in the harvestman. In addition, the “femur” domain of expression is enlarged and clearly extends beyond the position of the developing joint, suggesting another or additional function(s) than merely the development of the joint in this region of the appendages. The expression of this gene is thus not associated with the development of all joints, and data from other arthropods, including *Drosophila*, are currently not available.

The expression of *Parasteatoda* and *Phalangium unc4516* is conserved in the joints, suggesting a conserved role in joint development, at least in the group of arachnids to which harvestmen and spiders belong. Interestingly, orthologs of *unc4516* appear to be present in various groups of protostomian and deuterostomian animals, demonstrating that it was present in the last common ancestor of bilaterians. In none of these animals, however, *unc4516* has been studied thus far. The ancestral function of *unc4516* could be associated with the development of the brain because both spider paralogs and the harvestman *unc4516* gene are inter alia expressed in this structure (Figures 6, 10 and Appendix S7). Further gene expression studies, including representatives of all main groups of bilaterian animals, will be needed to address this question and the question of whether the expression and function of *unc4516* in the developing joints is conserved in Arthropoda as a whole.

In *Drosophila, al* is involved in the development of distal structures of the appendages, including the joints.^78^ In line with this, *al* is expressed in subsets of joints in the distal part of developing *Drosophila* appendages, and these patterns are conserved in other insects than *Drosophila* and a myriapod, corroborating the *Drosophila* data and thus suggesting a function in joint development in the distal part of the appendages.^50^, ^54^, ^79^ A previous study has shown that *al* is also expressed in some rings in the developing appendages of the spiders *Parasteatoda* and *Pholcus*.^31^ A slightly different pattern has been shown for the harvestman *Phalangium*, in which *al* is only expressed in the very tip of developing appendages and transiently in a single distal ring.^22^ It can, however, be assumed that *al* is involved in some form in arthropod appendage patterning and possibly also joint development.

The *omb* gene is a conserved factor of dorsal appendage patterning in arthropods including spiders, but there are no reports on an expression pattern in rings during appendage development and joint development.^44^, ^80^, ^81^, ^82^, ^83^ In both *Drosophila* and *Tribolium*, however, loss of *omb*, or *omb* and *dorsocross* (*doc*) (another related T‐box transcription factor encoding gene) leads to dorsal joint defects, likely because of the dorsal disturbance of Notch‐signaling and/or Decapentaplegic‐signaling.^83^, ^84^

The expression of *Parasteatoda* and *Phalangium unc8595* is conserved and restricted to the joints, strongly supporting a conserved role in joint development, at least in the group of chelicerates uniting harvestmen and spiders. The unclear orthology to genes in other groups of animals makes it impossible to draw further conclusions, but further research may reveal that *unc8595* could represent an arachnid‐specific appendage patterning gene.

The *trh* gene is a known factor of distal limb patterning and joint development in *Drosophila*,^78^ and loss of *trh* leads to some distal joint phenotypes in the developing legs.^85^ In the hemipteran *Oncopeltus*, *trh* is expressed in a few rings in the developing appendages.^86^ In spiders (including *Parasteatoda*) and a harvestman, expression of *trh* (*trh‐1* in spiders) in a few rings suggests a function in joint development in this group of arthropods as well.^31^, ^32^ The available data thus suggest that *trh* may indeed play a conserved role in arthropod joint development.

The *cll* gene of *Drosophila* is a known factor of distal appendage development,^78^ and in *Tribolium* expression in the tip of the appendages is conserved.^24^ Likewise, *cll* is expressed in the tips of the developing antennae and legs in the myriapod *Glomeris*.^50^ Expression of the single *cll* gene of the harvestman *Phalangium* is restricted to the distal region of the developing appendages,^22^, ^42^ and a recent study presented expression of *cll* in the tips of developing appendages of a sea spider.^22^ The *cll* genes (also named *Tlx*) of the spiders *Parasteatoda* and *Pholcus* have been studied before and are expressed in the distal region of the prosomal appendages including in rings.^31^, ^42^ Interestingly, RNAi‐mediated knockdown of *cll1* in *Parasteatoda* revealed phenotypes including the loss of the tarsal region (strong phenotype) or the fusion/loss of the joint between metatarsus and tarsus; unlike the phenotype in *Drosophila*, the claw, however, remained unaffected suggesting functional divergence although this remains to be tested because the second paralog was not knocked down in their experiments, and neither was a double knockdown of both paralogs performed.^31^ Either way, it appears that *cll* plays a conserved role in distal appendage development including distal joint development in arthropods, although this is not a general function in joint development.

## 4 FUTURE PERSPECTIVES

Given the paucity of comparative data on many of the identified C3‐cluster genes, additional studies are needed, including species of myriapods, crustaceans, and indeed *Drosophila*, to further track the evolutionary origin of the expression patterns discovered in *Parasteatoda* and *Phalangium*, especially for those of the genes that are possibly involved in joint development. In this context, it has to be kept in mind that the expression of genes at the place (or near the place) where the joints form does not necessarily mean that these genes are actively involved in the development of the joints as morphological structures sensu stricto (a hinge‐like structure). Their expression (and thus function) could indeed also be linked to structures that are associated with the joints, such as specific sensory organs, or they could be involved in podomere growth, or both joint development and growth, as described for *Drosophila*.^87^ Likewise, the fate and final position of the cells that express the newly identified candidate genes are unclear given the massive morphogenetic changes during arthropod joint development.^88^, ^89^, ^90^ Therefore, it would be very helpful to also obtain functional data about the newly discovered/identified appendage and joint patterning genes in *Parasteatoda* and/or *Phalangium*. A feasible way to address this question is RNA interference (RNAi), a method that is well‐established in both the spider *Parasteatoda* and the harvestman *Phalangium*.^62^, ^91^ The advantage of the latter species is the apparent lack of paralogs that, in some cases, could complicate functional analysis due to redundancy effects. Another method that could be applied and that would better illustrate the relative position of gene expression of the new candidate genes with respect to known joint‐patterning genes is di‐ or multichromatic double in situ hybridization, including the recently established hybridization‐chain reaction (HCR).^92^

## 5 EXPERIMENTAL PROCEDURES

### 5.1 Research animals, embryos and handling of genetic material

Embryos of the spider *Parasteatoda* stem from our own culture in Uppsala and were treated as described in Prpic et al.^93^ Fertilized females of *Phalangium* were collected during the autumn months around the paleontological museum in Uppsala and kept in plastic containers provided with a dish of sphagnum moss (for egg deposition). After egg deposition, the animals were released into the wild again. Embryos of the harvestman *Phalangium* were obtained and treated as described in Janssen et al.^94^ The embryonic stages are defined after (*Parasteatoda*)^95^ and (*Phalangium*).^96^

For all investigated species, total RNA was isolated from embryos of different stages using TRIZOL (Invitrogen). Subsequently, mRNA was isolated from the total RNA using the Dynabeads mRNA Purification Kit (Invitrogen). The purified mRNA was reverse transcribed into cDNA using the Superscript II first strand synthesis system for RT‐PCR (Invitrogen). Gene fragments were isolated using gene specific primers (based on the sequenced genomes of the spider *Parasteatoda* ^41^, ^97^ (PRJNA167405; PRJNA934108)), and the sequenced embryonic transcriptomes of the harvestman *Phalangium* ^76^ (SRX9813550, SRX450969) with T7‐promoter overhangs on the reverse primers (Appendix S1).

### 5.2 Probe synthesis and in situ hybridization

All probes were synthesized using T7‐RNA polymerase (Roche) and Dig‐labeled rNTPs (Roche). Purified PCR products (QIAquick PCR purification kit, QIAGEN) were used as templates for probe synthesis. Probes were synthesized for 2–4 h at 37°C and purified using the RNeasy Mini Kit (QIAGEN). All in situ hybridizations were performed using the same standardized protocol. The latest version of this protocol is deposited in Appendix S1 (Appendix S2).

### 5.3 Phylogenetic analyses

All phylogenetic analyses were essentially performed as described in Panara et al.^98^ using MRBAYES.^99^ We aligned available protein sequences using T‐Coffee^100^ and manually edited the alignments. Gene topology was calculated by applying 0.5 (Odd‐tree), 1 (Spz‐tree), and 2 (Klf‐tree) million cycles in the MCMCMC analysis (with a chain‐heating temperature of 0.2 and sampling of chains every 200 cycles; 25% of samples were applied as burn‐in). For all trees, clade support was determined with posterior probabilities in MRBAYES. All trees are midpoint rooted.

## CONFLICT OF INTEREST STATEMENT

The authors declare no conflicts of interest.

## Supporting information


**Appendix S1:** C3‐markers, identifiers, and primers.

**Appendix S2:** Odd‐Tree and Alignment. The blue asterisk marks the C3‐marker gene, and black asterisks mark paralogs of this gene investigated in this paper. The orange asterisk marks the *Phalangium* ortholog. Species abbreviations: Cs, *Cupiennius salei*; Dm, *Drosophila melanogaster*; Po, *Phalangium opilio*; Pt, *Parasteatoda tepidariorum*; Tc, *Tribolium castaneum*.

**Appendix S3:** Spz‐Tree and Alignment. The blue asterisk marks the C3‐marker gene, and the black asterisks marks a paralog of this gene investigated in this paper. The orange asterisk marks the *Phalangium* ortholog. Species abbreviations: Dm, *Drosophila melanogaster*; Po, *Phalangium opilio*; Pt, *Parasteatoda tepidariorum*; Tc, *Tribolium castaneum*.

**Appendix S4:** Klf‐Tree and Alignment. The blue asterisk marks the C3‐marker gene and the orange asterisk marks the *Phalangium* ortholog. Species abbreviations: Dm, *Drosophila melanogaster*; Hs, *Homo sapiens*; Po, *Phalangium opilio*; Pt, *Parasteatoda tepidariorum*; Tc, *Tribolium castaneum*.

**Appendix S5:** Figure S1 – Late expression of *Parasteatoda Dll* and *Dfd‐B* and comparison of *dysf* and *Tmtc‐like1*. Expression of *Dll* (A), *Dfd‐B* (B) and comparison of *Tmtc‐like1* and *dysf* expression in the leg (C). Note the faint rings of expression within the otherwise ubiquitous expression domains of *Dll* and *Dfd‐B* in the appendages. Also note the missing domains of *Tmtc‐like1* compared to the joint marker *dysf* (marked with red asterisks). Abbreviations: see Figure 2.

**Appendix S6:** Figure S2 – Paralogs of C3‐cluster genes I. Expression of *odd‐r1* (A–F) and *odd‐r2* (G–L). In all panels, anterior is to the left except for panels showing dissected head lobes (anterior up) and appendages (proximal up). Panel E shows a dissected pedipalp. Panels F, K and L show dissected legs. Panels A′‐C′, G′–I′, and J′ represent SYBR‐green staining of corresponding embryos. Abbreviations: see Figure 2.

**Appendix S7:** Figure S3 – Paralogs of C3‐cluster genes II. Expression of *sobp2* (A–D), *svp2* (E–M), *Tox‐like2* (N–V), and *unc4516.2* (W–a). In all panels, anterior is to the left except for panels showing dissected head lobes (anterior up) and appendages (proximal up). Panels D shows a dissected head lobe with chelicerae and pedipalps. Panels H and Q show dissected head lobes with chelicerae. The asterisk in panel D marks expression in the head lobes. Panels L, U, and Z show dissected pedipalps. Panels M, A, and a show dissected legs. The filled circles in panels F, G, I, J, L, and M mark expression in the tips of the appendages. The asterisk in panel K marks dorsal expression at the interface between the pro‐ and the opisthosoma. The asterisk in panel O marks faint expression in the ventral nervous system. Abbreviations: see Figure 2.

**Appendix S8:** In situ hybridization protocol.

## Data Availability

The data that supports the findings of this study are available in the supplementary material of this article.
