# Supplementary figures and images for: Single‐cell sequencing reveals potential novel insights into appendage‐patterning and joint‐development in a spider
Source: Dev Dyn. 2025 Aug 7;255(7):671–97. doi: 10.1002/dvdy.70069 (PMC13353597; doi:10.1002/dvdy.70069)

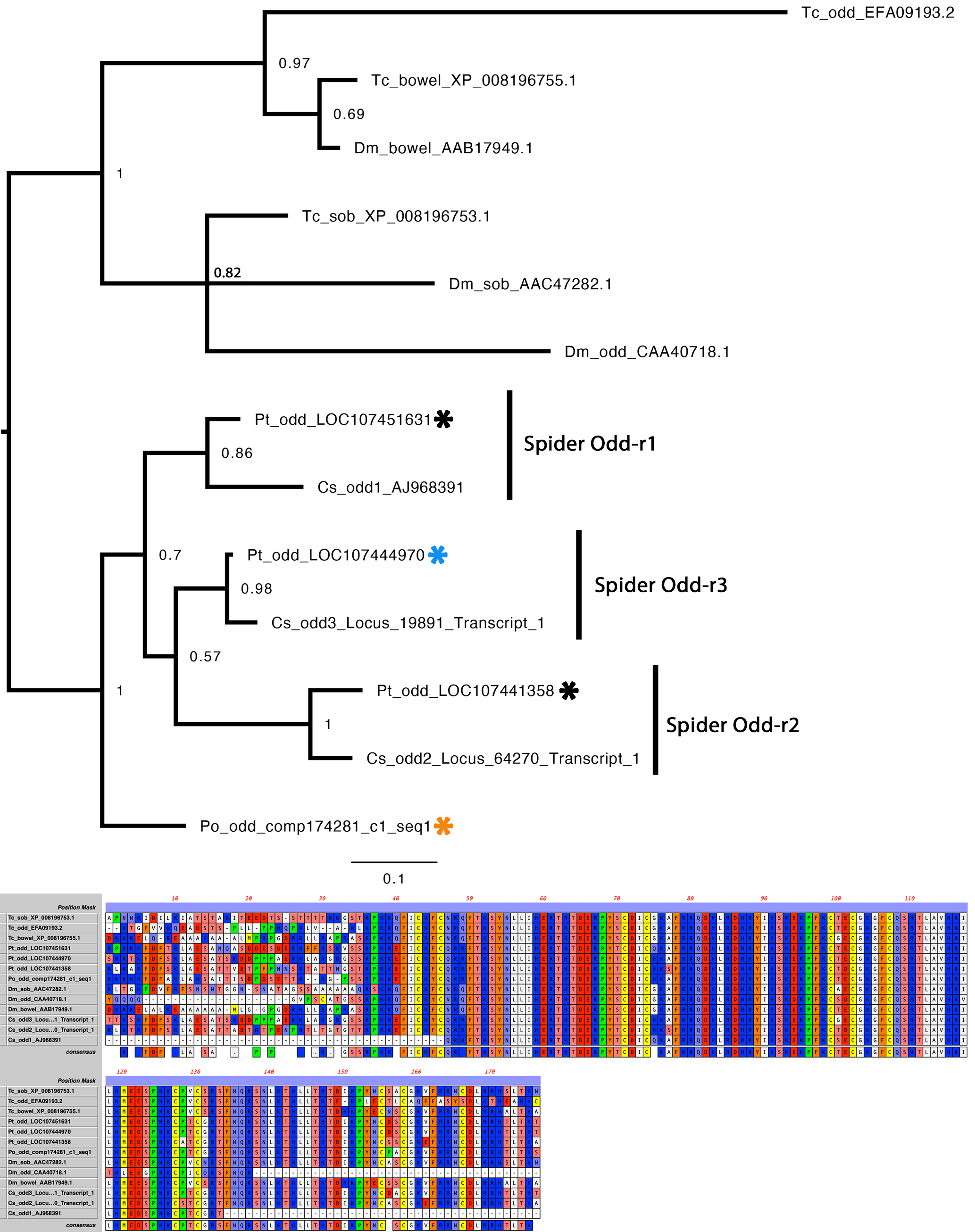

Supplement: Supplementary file 2 — Appendix S2: Odd‐Tree and Alignment. The blue asterisk marks the C3‐marker gene, and black asterisks mark paralogs of this gene investigated in this paper. The orange asterisk marks the Phalangium ortholog. Species abbreviations: Cs, Cupiennius salei; Dm, Drosophila melanogaster; Po, Phalangium opilio; Pt, Parasteatoda tepidariorum; Tc, Tribolium castaneum. [file DVDY-255-671-s001.tif]

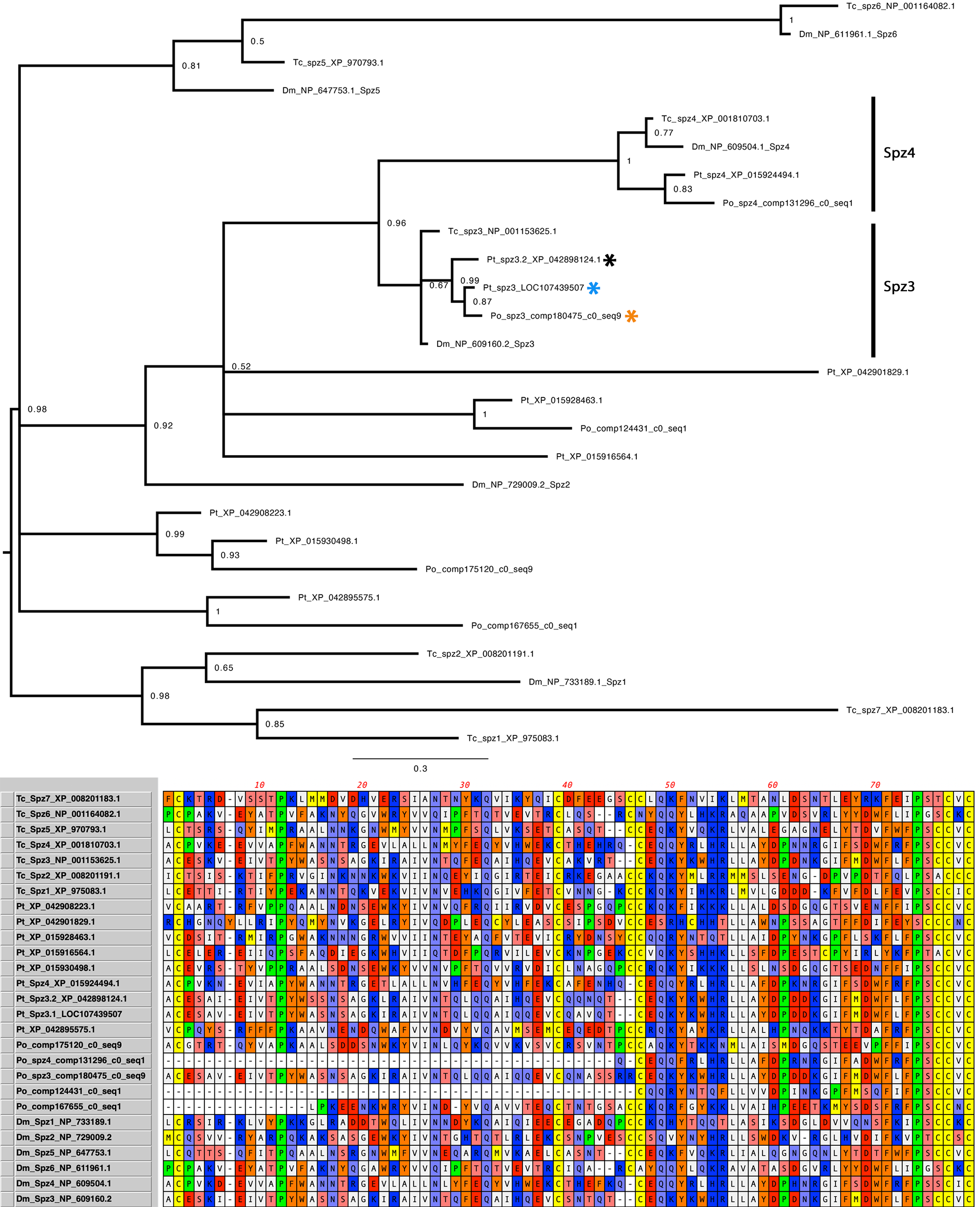

Supplement: Supplementary file 3 — Appendix S3: Spz‐Tree and Alignment. The blue asterisk marks the C3‐marker gene, and the black asterisks marks a paralog of this gene investigated in this paper. The orange asterisk marks the Phalangium ortholog. Species abbreviations: Dm, Drosophila melanogaster; Po, Phalangium opilio; Pt, Parasteatoda tepidariorum; Tc, Tribolium castaneum. [file DVDY-255-671-s006.tif]

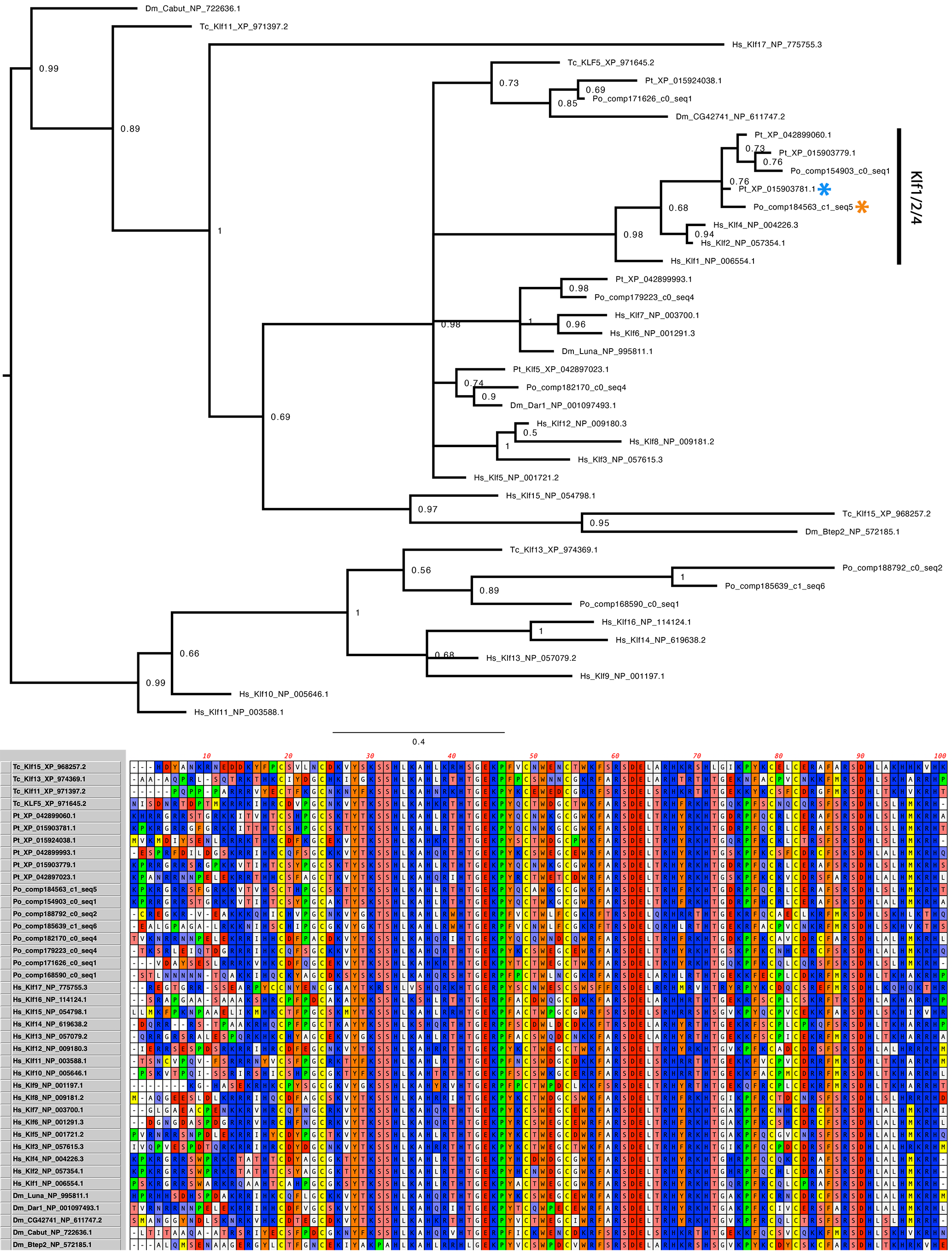

Supplement: Supplementary file 4 — Appendix S4: Klf‐Tree and Alignment. The blue asterisk marks the C3‐marker gene and the orange asterisk marks the Phalangium ortholog. Species abbreviations: Dm, Drosophila melanogaster; Hs, Homo sapiens; Po, Phalangium opilio; Pt, Parasteatoda tepidariorum; Tc, Tribolium castaneum. [file DVDY-255-671-s005.tif]

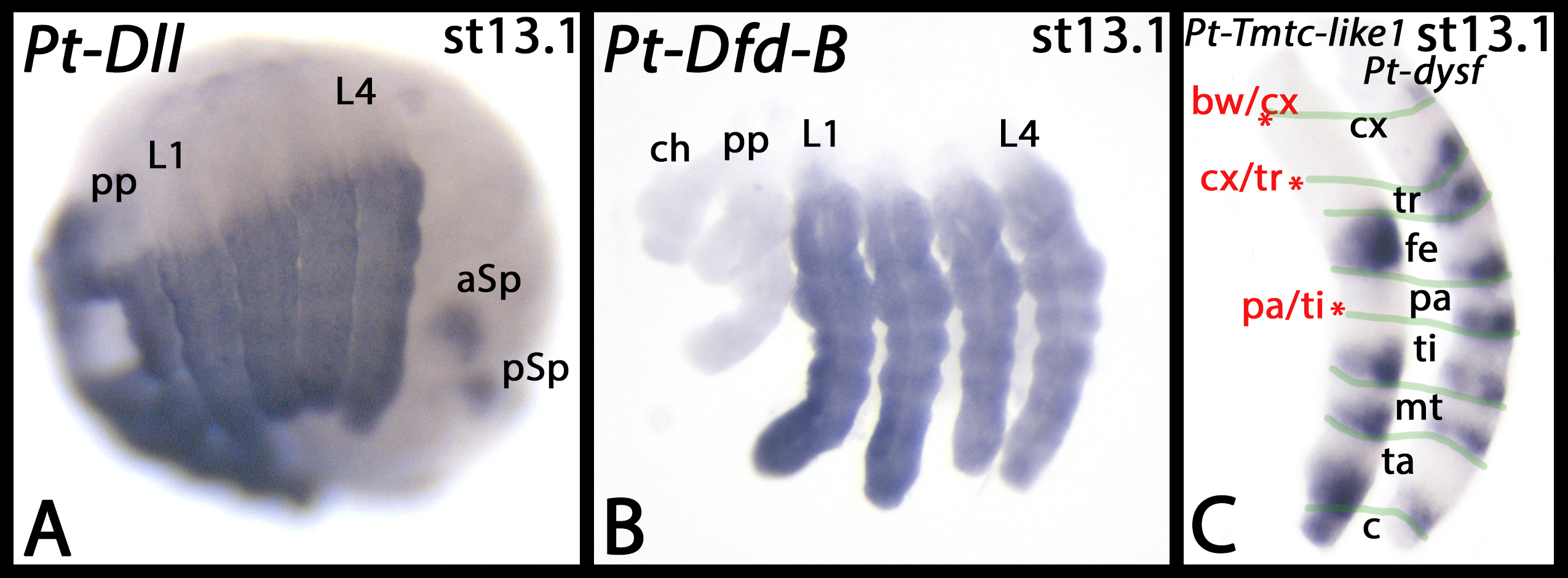

Supplement: Supplementary file 5 — Appendix S5: Figure S1 – Late expression of Parasteatoda Dll and Dfd‐B and comparison of dysf and Tmtc‐like1. Expression of Dll (A), Dfd‐B (B) and comparison of Tmtc‐like1 and dysf expression in the leg (C). Note the faint rings of expression within the otherwise ubiquitous expression domains of Dll and Dfd‐B in the appendages. Also note the missing domains of Tmtc‐like1 compared to the joint marker dysf (marked with red asterisks). Abbreviations: see Figure 2. [file DVDY-255-671-s004.tif]

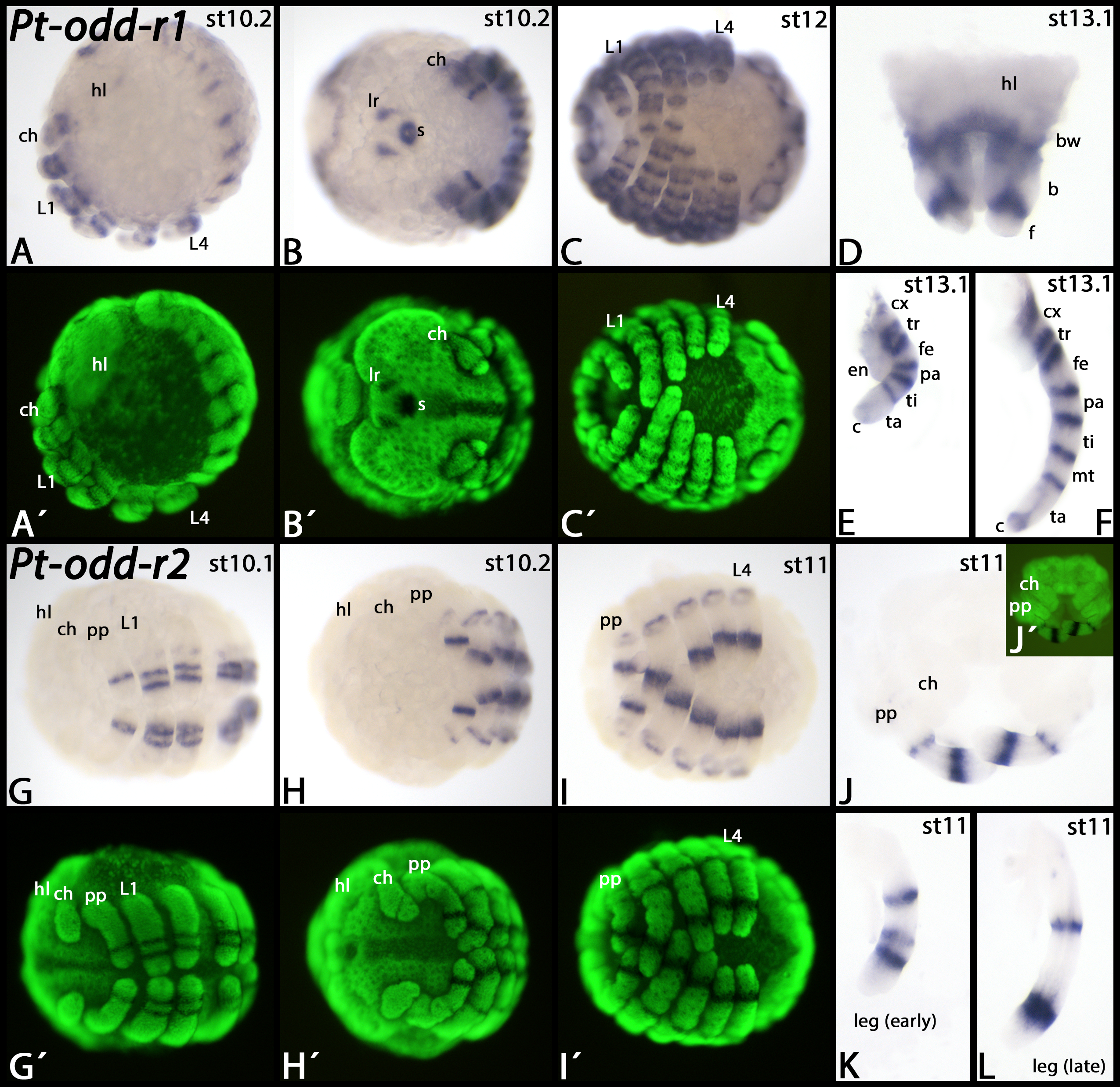

Supplement: Supplementary file 6 — Appendix S6: Figure S2 – Paralogs of C3‐cluster genes I. Expression of odd‐r1 (A–F) and odd‐r2 (G–L). In all panels, anterior is to the left except for panels showing dissected head lobes (anterior up) and appendages (proximal up). Panel E shows a dissected pedipalp. Panels F, K and L show dissected legs. Panels A′‐C′, G′–I′, and J′ represent SYBR‐green staining of corresponding embryos. Abbreviations: see Figure 2. [file DVDY-255-671-s008.tif]

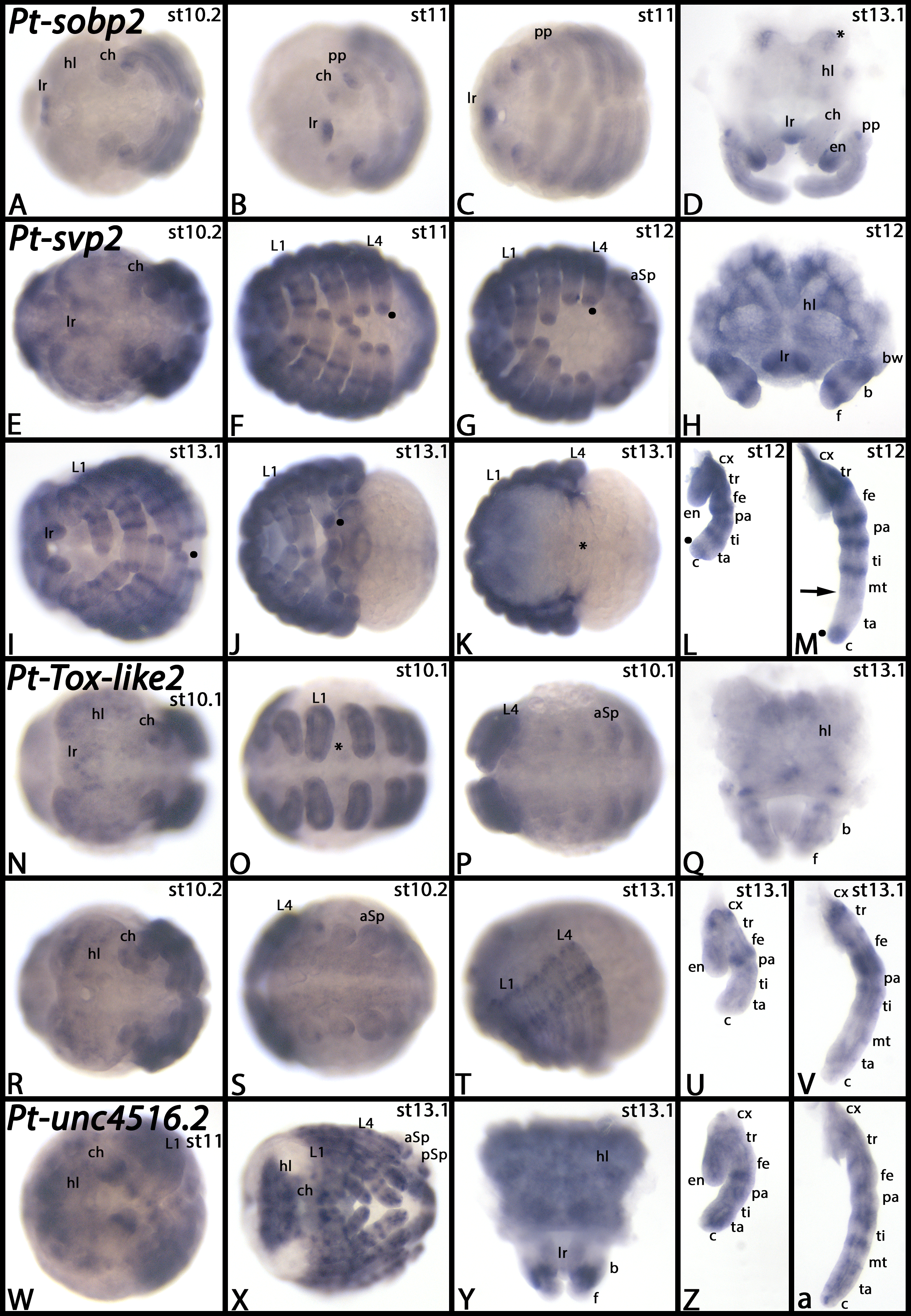

Supplement: Supplementary file 7 — Appendix S7: Figure S3 – Paralogs of C3‐cluster genes II. Expression of sobp2 (A–D), svp2 (E–M), Tox‐like2 (N–V), and unc4516.2 (W–a). In all panels, anterior is to the left except for panels showing dissected head lobes (anterior up) and appendages (proximal up). Panels D shows a dissected head lobe with chelicerae and pedipalps. Panels H and Q show dissected head lobes with chelicerae. The asterisk in panel D marks expression in the head lobes. Panels L, U, and Z show dissected pedipalps. Panels M, A, and a show dissected legs. The filled circles in panels F, G, I, J, L, and M mark expression in the tips of the appendages. The asterisk in panel K marks dorsal expression at the interface between the pro‐ and the opisthosoma. The asterisk in panel O marks faint expression in the ventral nervous system. Abbreviations: see Figure 2. [file DVDY-255-671-s002.tif]
