# Supplementary material for: Single‐cell sequencing reveals potential novel insights into appendage‐patterning and joint‐development in a spider
Source: Dev Dyn. 2025 Aug 7;255(7):671–97. doi: 10.1002/dvdy.70069 (PMC13353597; doi:10.1002/dvdy.70069)
Supplement: Supplementary file 8 — Appendix S8: In situ hybridization protocol. [file DVDY-255-671-s007.docx]

**Whole mount in-situ hybridization (*Parasteatoda*, *Cupiennius*, *Pardosa*, *Pholcus*, *Acanthoscurria*, *Ischnothele*, *Phalangium*, *Drosophila*, *Tribolium, Pyrrhocoris, Glomeris* and *Euperipatoides*)**

**_______________________________________________________________________day 1**

**Rehydration**

❑ Return embryos to RT

❑ Wash 5 min in 50% MeOH in PBS-T

❑ Wash 5 min in 25% MeOH in PBS-T

❑ ❑ ❑ Wash three times in PBS-T for 10 min each

**Prehybridization**

❑ Wash embryos once in 1:1 PBS-T:HYB-B for 10 min (first add 500µl PBS-T, then 500µl HYB-B (mix gently by inverting))

❑ Replace and incubate embryos in 500μl HYB-B for 5 min @ 65°C

❑ Replace HYB-B with 500μl of prewarmed HYB-A

❑ Prehybridize @ 65°C for a minimum of 2h in HYB-A

**Hybridization**

❑ Remove HYB-A from the embryos without letting them touch air

❑ Add 50µl of prewarmed fresh HYB-A

❑ Add 2-3 µL anti-sense RNA probe, mix gently, and incubate o/n @ 65°C

**________________________________________________________________day 2**

**Probe removal**

❑ Add 500μl prewarmed HYB-B, incubate 15 min @ 65°C

❑ Replace with 1:1 mixture of HYB-B and 2XSSC incubate 15 min @ 65°C (500µl each, HYB-B first)

❑ Wash with 2xSSC for 15 min @ 65°C

❑ ❑ ❑ Wash three times with PBS-T for 10 min each @ room temperature (**RT)**

❑ Incubate in blocking buffer for at least 2 h @ **RT**

**Detection**

❑ Prepare 1:2000 dilution of AP conjugated DIG-antibody in 1 ml Blocking buffer

❑ Incubate embryos with DIG-antibody mixture for at least 1.5 h

❑ ❑ ❑ ❑ Wash four times in PBS-T for 10 min each

❑ Incubate in PBS-T o/n @ 4°C

**________________________________________________________________day 3**

**Staining**

❑ ❑ ❑ Wash three times with PBS-T for 5 min

❑ ❑ ❑ Wash three times with AP staining buffer for 5 min

❑ Centrifuge BM purple for 1min at ca. 7500 RPM. Use supernatant for staining. Incubate with BM purple staining solution and monitor color development under the microscope (stain in the dark).

❑ ❑ ❑ Stop staining reaction by washing 3 times with AP staining buffer (pH7.4) (= STOP solution) for 5 min each

❑ Counterstain with CYBR Green 1:10000 for 30min (in the dark)

❑ ❑ ❑ Remove solution by 3 times washing for 10 min in STOP solution

❑ Store @ 4°C. Do NOT freeze!

**­­­10x PBS stock**

1.37 M NaCl

27 mM KCl

100 mM Na2HPO4

adjust pH to 7.4

**PBS-T**

1 x PBS

0.1 % Tween-20

**SSC**

saline-sodium citrate buffer

**HYB-B**

50% formamide

25% 20x SSC, pH 7.0

0.1% Tween-20

adjust pH to 6.5

**HYB-A**

1x HYB-B

125 µl of 20 mg/ml heparin

5% Dextran Sulfate Salt (Sigma) (2.5g in 50 ml)

0.01 mg/ml yeast RNA (or 250 µl 20mg/ml tRNA)

0.4 mg/ml sonicated salmon sperm DNA

store @ -20°C

**Blocking buffer**

1x PBS-T

1% BSA

2% Sheep serum

**AP staining buffer**

100 mM Tris pH 9.5

150 mM NaCl

10 mM MgCl2

0.1% Tween-20

**STOP solution**

Same as AP staining buffer, BUT pH 7.4
